# Elucidating the origins of phycocyanobilin biosynthesis and phycobiliproteins

**DOI:** 10.1073/pnas.2300770120

**Published:** 2023-04-18

**Authors:** Nathan C. Rockwell, Shelley S. Martin, J. Clark Lagarias

**Affiliations:** ^a^College of Biological Sciences, Department of Molecular and Cell Biology, University of California at Davis, Davis, CA 95616

**Keywords:** phytochrome, cyanobacteriochrome, phylogenetic analysis, phycobilisome, light harvesting antennae

## Abstract

Terrestrial ecosystems and human societies depend on oxygenic photosynthesis, which began to reshape our atmosphere approximately 2.5 billion years ago. The earliest known organisms carrying out oxygenic photosynthesis are the cyanobacteria, which use large complexes of phycobiliproteins as light-harvesting antennae. Phycobiliproteins rely on phycocyanobilin (PCB), a linear tetrapyrrole (bilin) chromophore, as the light-harvesting pigment that transfers absorbed light energy from phycobilisomes to the chlorophyll-based photosynthetic apparatus. Cyanobacteria synthesize PCB from heme in two steps: A heme oxygenase converts heme into biliverdin IXα (BV), and the ferredoxin-dependent bilin reductase (FDBR) PcyA then converts BV into PCB. In the current work, we examine the origins of this pathway. We demonstrate that PcyA evolved from pre-PcyA proteins found in nonphotosynthetic bacteria and that pre-PcyA enzymes are active FDBRs that do not yield PCB. Pre-PcyA genes are associated with two gene clusters. Both clusters encode bilin-binding globin proteins, phycobiliprotein paralogs that we designate as BBAGs (bilin biosynthesis-associated globins). Some cyanobacteria also contain one such gene cluster, including a BBAG, two V4R proteins, and an iron–sulfur protein. Phylogenetic analysis shows that this cluster is descended from those associated with pre-PcyA proteins and that light-harvesting phycobiliproteins are also descended from BBAGs found in other bacteria. We propose that PcyA and phycobiliproteins originated in heterotrophic, nonphotosynthetic bacteria and were subsequently acquired by cyanobacteria.

## Significance

Human societies rely on photosynthesis to provide the food we eat and the oxygen we breathe. All photosynthetic organisms use light-harvesting antennae to capture enough light to sustain growth. Oxygen-producing photosynthesis evolved 2.5 billion years ago in cyanobacteria, whose antennae consist of phycobilisomes (complexes of phycobiliproteins). Phycobilisomes all require phycocyanobilin (PCB) both to harvest light and to relay light energy to the sites of photosynthesis. In this work, we demonstrate that PcyA, the cyanobacterial enzyme that makes PCB, evolved from enzymes that catalyze similar reactions in nonphotosynthetic bacteria. The acquisition of these enzymes and phycobiliprotein precursors enabled evolution of the phycobiliprotein antenna systems. Hence, this work illuminates a key innovation in the evolution of oxygenic photosynthesis.

Human societies depend on oxygenic photosynthesis to provide food, fodder, and a breathable atmosphere. Modern oxygenic photosynthetic organisms are either cyanobacteria or acquired this ability from cyanobacteria via endosymbiosis, preserving a core photosynthetic apparatus across more than 2.5 billion years of evolution. This apparatus centers on photosystems I and II, large membrane-bound protein complexes, and a special pair of chlorophyll (Chl) molecules at the active site for oxygen evolution. This arrangement provides the ability to use light to split water, but the cross-section for photon absorption by the special pair is too small to sustain robust photoautotrophy. Therefore, photosynthetic organisms have evolved large antenna complexes to increase the photosynthetic cross-section and power photosynthesis via energy transfer (ET).

Different photosynthetic organisms use different antennae. Cyanobacteria use phycobilisomes, large protein complexes comprised of an array of phycobiliproteins and linkers with bound linear tetrapyrrole (bilin) chromophores that are synthesized via the same trunk pathway that makes Chl (*SI Appendix*, Fig. S1*A*). This light-harvesting scheme offers great flexibility for cyanobacteria, because they can readily add different phycobiliproteins tuned to harvest different wavelengths of light to facilitate growth in environments that are depleted of photosynthetically optimal red light (1, 2). For example, under green light, some cyanobacteria induce synthesis of phycoerythrin and phycoerythrobilin (PEB) to capture light that is poorly resonant with Chl (*SI Appendix*, Fig. S1 *B* and *C*). This strategy leverages ET from higher-energy phycoerythrin through red-absorbing phycocyanin and allophycocyanin, ultimately transferring energy to Chl (*SI Appendix*, Fig. S1*B*). This ET funnel relies on covalently bound phycocyanobilin (PCB, *SI Appendix*, Fig. S1*C*), the chromophore for phycocyanin and allophycocyanin. PCB is thus critical for cyanobacterial light-harvesting strategies. In addition to its role in photosynthesis, PCB provides photoactive chromophore precursors for cyanobacterial phytochrome and cyanobacteriochrome (CBCR) photoreceptors (3, 4). PCB also binds to GUN4, an accessory subunit of magnesium chelatase, thereby providing a photoprotective role for the enzyme catalyzing the committed step in Chl biosynthesis (5, 6). PCB is thus critical for both light harvesting and Chl biosynthesis in cyanobacteria, making PCB biosynthesis a critical step in the rise of oxygenic photosynthesis and the ultimate oxygenation of our atmosphere.

The origins of bilin-based light harvesting are poorly understood. Synthesis of the cyanobacterial bilins PCB and PEB takes multiple steps after the synthesis of heme (7, 8). A heme oxygenase (HO) first converts heme to biliverdin IXα (BV, *SI Appendix*, Fig. S1*A*). One or two ferredoxin-dependent bilin reductases (FDBRs) then convert BV into different bilins in different organisms (*SI Appendix*, Fig. S1*C*). There is thus a “chicken-and-egg” question for evolution of cyanobacterial photosynthesis: Synthesis of light-harvesting bilins requires abundant oxygen for HO turnover, but oxygen would only be present in significant quantities once light could be efficiently harvested. One of the major open questions is the origin of the last step in PCB biosynthesis, catalyzed by the FDBR PcyA (*SI Appendix*, Fig. S1*C*). PcyA converts BV into PCB in a 4-electron reduction that proceeds via the intermediate 18^1^,18^2^–dihydrobiliverdin (18^1^,18^2^–DHBV, *SI Appendix*, Fig. S1*D*), itself a known chromophore precursor for some CBCRs (9). Unlike genes for PEB biosynthesis, *pcyA* appears to be an essential gene in cyanobacteria (10); however, its origin remains an enigma. Whereas *pcyA* genes encoding active enzymes are also found in some α-proteobacteria (11, 12) and in green algae (13), neither seem to be descended from extant cyanobacterial *pcyA* sequences (7, 12, 14). The origin of the light-harvesting phycobiliproteins, members of the globin superfamily, is similarly obscure.

The present work was undertaken to provide insight into the origins of PcyA and the phycobiliproteins. Phylogenetic analyses demonstrate that PcyA is descended from pre-PcyA proteins associated with conserved gene clusters in diverse bacteria also found in some modern cyanobacteria. Pre-PcyA-associated genes include distant phycobiliprotein homologs that we designate as biosynthesis-associated globins (BBAGs), a broad family also including extant phycobiliproteins. Through reconstitution of pre-PcyA activity in *Escherichia coli,* we demonstrate that pre-PcyA sequences are functional FDBR enzymes and that BBAGs are self-assembling biliproteins. Based on these findings, we conclude that PCB biosynthesis and bilin-binding globins were acquired by cyanobacteria from heterotrophic bacteria and were subsequently tailored for light harvesting during evolution of oxygenic photosynthesis.

## Results

### Phylogenetic Analysis Identifies Pre-PcyA Proteins in Heterotrophic Bacteria.

**Fig. 1.**
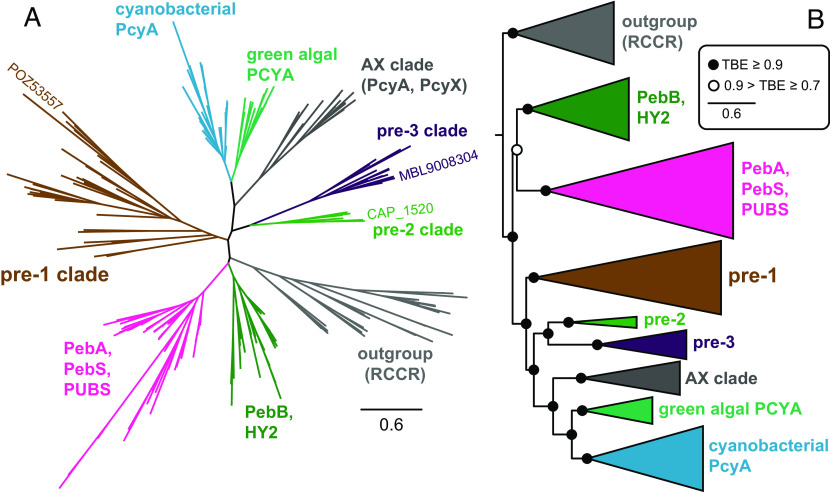
Evolution of PcyA from pre-PcyA sequences. A maximum-likelihood phylogenetic tree for FDBRs was inferred as described in *Methods* and in *SI Appendix*, Table S2. The complete tree is presented in unrooted (*A*) and simplified (*B*) views, with previously reported enzymatic activities indicated as in *SI Appendix*, Fig. S1. The positions of pre-PcyA proteins characterized in this study are shown in panel *A*. For panel *B*, the root is placed between the outgroup and all lineages containing known FDBRs. Pre-PcyA lineages are indicated. The AX clade comprises PcyA from α-proteobacteria and PcyX from phages. The complete tree is shown in *SI Appendix*, Figs. S2 and S3, using the root placement of panel *B*. Support was calculated using the transfer bootstrap expectation value (TBE) as described in the Methods.

Our previous analysis of FDBR diversification (7) identified a pair of sequences from *Chondromyces* spp. that were weakly related to PcyA sequences, along with a group of sequences from Candidatus Schekmanbacteria that seemed to fall in between FDBRs and the distantly related red Chl catabolite reductases (RCCRs). More recently, we observed that related sequences have appeared in diverse metagenomic studies, allowing us to detect a much broader range of candidate sequences by using these sequences as search queries. As with the *Chondromyces* and Ca. Schekmanbacteria proteins, these sequences were not found in oxygenic photosynthetic organisms. We therefore revisited our previous phylogenetic analysis, including such sequences along with known FDBR families. We also included RCCRs and related sequences as an outgroup (7, 15). The resulting tree is shown in Fig. 1 and *SI Appendix*, Figs. S2 and S3.

As with previous analyses (7, 16), the outgroup defines a clade of known FDBRs that is split into two branches relative to the RCCR outgroup (Fig. 1*B*). Sequences from Ca. Schekmanbacteria clearly fall within the outgroup (*SI Appendix*, Fig. S2). One FDBR branch (*SI Appendix*, Fig. S2) comprises a lineage including the known FDBRs such as PebA, PebS, and PUBS (16–19) and another including PebB and HY2 (14, 16, 20, 21). The other branch (*SI Appendix*, Fig. S3) contains PcyA and PcyX sequences (11, 12, 16, 22). This branch also contains early-branching lineages of candidate FDBRs that we designate as “pre-PcyAs.” Consistent with previous analyses (7, 12), PcyA and PcyX sequences form a clade with three lineages: an AX lineage comprising α-proteobacterial PcyA and phage PcyX; a “green” lineage comprising PCYA from prasinophyte and chlorophyte algae and from the dinoflagellate genera *Lepidodinium* and *Karenia*; and a mostly cyanobacterial PcyA lineage that also includes PcyA sequences from cyanophages and from glaucophyte, rhodophyte, and cryptophyte algae.

Within cyanobacterial PcyA sequences, the first two branches are sequences from Gloeobacterales and then Thermostichales (*SI Appendix*, Fig. S3), consistent with the early branching pattern of these cyanobacterial lineages (23–25). The pre-PcyA lineages are basal to PcyA and PcyX relative to the outgroup and do not include cyanobacterial sequences. Pre-PcyA sequences form two clades. We designate the earliest branching clade as pre-1. The second clade was itself split into two branches, and the proteins in these branches exhibited characteristic differences at potential catalytic residues (*SI Appendix*, Fig. S3) and distinct chromosomal contexts (see below). We therefore designate these as pre-2 and pre-3. This analysis thus demonstrates that PcyA arose from a pre-PcyA ancestor that was subsequently acquired by cyanobacteria.

We observed variation in the distribution and composition of the three pre-PcyA lineages (*SI Appendix*, Fig. S3). Pre-3 sequences reported to date are exclusively found in metagenome-assembled genomes (MAGs), whereas both pre-1 and pre-2 are also found in cultured organisms (*SI Appendix*, Fig. S3). Pre-1 is apparently more widespread, with examples from Chloroflexi, β- and γ-proteobacteria, Acidobacteria, Planctomycetes, and other bacterial lineages. Pre-2 and pre-3 are largely confined to δ-proteobacteria and in particular to Myxococcales, with the previously noted sequences from the Myxococcal genus *Chondromyces* belonging to pre-2 (*SI Appendix*, Fig. S3). The only extant pre-3 sequences not found in δ-proteobacteria are one sequence apiece from Melainabacteria and Sericytochromatia. Unlike proteobacteria, both Sericytochromatia and Melainabacteria are thought to be nonphotosynthetic lineages relatively closely related to cyanobacteria (26–30). However, in contrast to bacterial lineages more closely related to cyanobacteria, proteobacteria are known to contain examples of pre-1, of pre-2, of pre-3, and of active PcyA from the AX clade [*SI Appendix*, Fig. S3; (11, 12)].

We also observed systematic variation in potential active site residues of pre-PcyA lineages by comparison to PcyA. PcyA enzymes contain conserved Asp, His, and Glu residues that are critical for catalysis [Glu76, His88, and Asp105 in the *Synechocystis* enzyme: (31, 32)]. Analysis of the multiple sequence alignment indicated that these residues were absent in pre-1 (*SI Appendix*, Fig. S3). By contrast, Asp105 is present in pre-2 and pre-3, but His88 is not: pre-2 sequences instead contain an Asn residue, and pre-3 sequences contain a Thr residue. Glu76 also is absent in pre-PcyA sequences and in PcyX. These conclusions were further supported by examination of homology models of selected pre-PcyA proteins studied here (*SI Appendix*, Fig. S4 and see below) and of predicted pre-PcyA structures from the AlphaFold database [(33); *SI Appendix*, Fig. S5]. Extant pre-2 and pre-3 enzymes thus have active sites that seemingly more closely resemble that of PcyA than do those of pre-1, but key residues for PCB biosynthesis are apparently still missing. This analysis demonstrates that PcyA in oxygenic photosynthetic organisms is derived from pre-PcyA proteins found in heterotrophic bacteria.

### Pre-PcyA Proteins Are Active FDBRs.

To test whether pre-PcyA proteins are active FDBRs, we sought to reconstitute activity in *E. coli*. In this approach, an HO and FDBR were coexpressed with a bilin-binding phytochrome or CBCR reporter that has been previously examined with an established bilin coexpression system (34, 35). For example, the cyanobacterial phytochrome Cph1 can incorporate PCB or PΦB (36, 37), allowing its use to identify the reaction product of HY2 from *Klebsormidium flaccidum* (14). This approach can result in poor chromophorylation, so we first developed an improved coexpression system (*SI Appendix*, Fig. S6*A*). To do so, we noted that a restriction site introduced for characterization of KflaHY2 fortuitously improves homology to a sequence recently reported to improve protein expression in pET28 expression plasmids [*SI Appendix*, Fig. S6*B*: (38)]. We therefore designed an alternative PcyA (alt-PcyA) construct more closely matching this sequence. This construct was combined with *Synechocystis* ho1 (*sll1184*) or with the POZ53545 HO from *Methylovulum psychrotolerans* Sph1, recently reported to improve coproduction of BV in *E. coli* [*SI Appendix*, Fig. S6*A*; (39)]. Each plasmid was tested by coexpression with the CBCR Ava_3771. Ava_3771 incorporated a mixture of bilins when coexpressed with ho1 and PcyA (40) and bound BV when expressed with POZ53545 alone (*SI Appendix*, Fig. S6*C*). The combination of POZ53545 with the alt-PcyA construct gave both the highest observed chromophorylation (*SI Appendix*, Table S1) along with a substantial increase in PCB incorporation (*SI Appendix*, Fig. S6 *C*–*E*), indicating superior PcyA expression and/or function. We therefore used this plasmid, Spam-545/alt (*SI Appendix*, Fig. S6*A*), as a starting point for testing pre-PcyA proteins for FDBR activity.

**Fig. 2.**
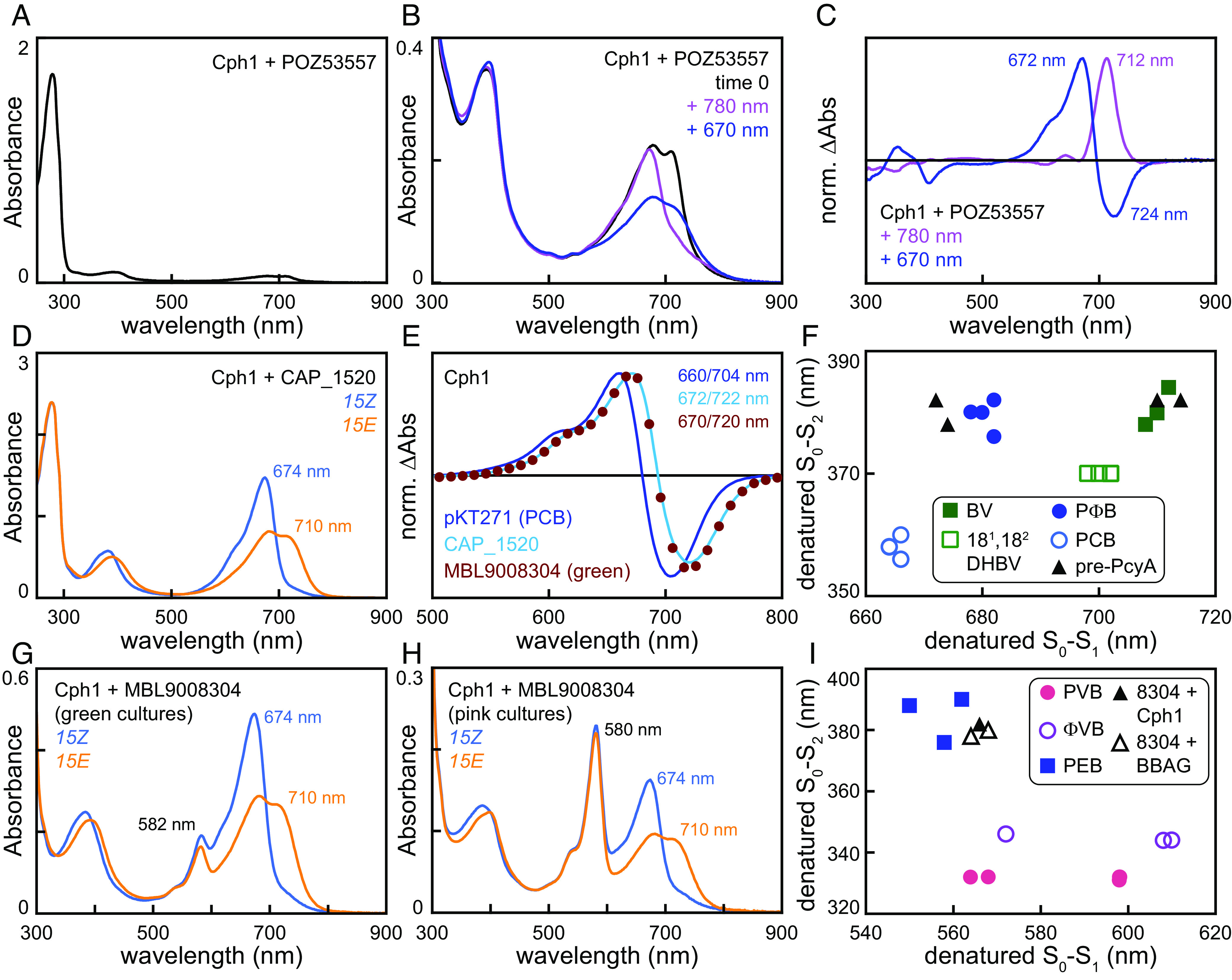
Pre-PcyA proteins are functional FDBRs. Bilin biosynthesis plasmids were recombinantly coexpressed with bilin-accepting photoreceptors in *E. coli.* (*A*) Cyanobacterial phytochrome Cph1 was coexpressed with the pre-1 FDBR POZ53557 (plasmid Spam-545/alt557: *SI Appendix*, Fig. S6*A*), purified, and characterized using absorption spectroscopy. (*B*) The same preparation as in panel A is shown at higher concentration. Black, material after dialysis (equivalent to panel *A*); mauve, after illumination to completion with 780 nm light; dark blue, after illumination to completion with 670 nm light. (*C*) Normalized photochemical difference spectra are shown for the spectra in panel B with 780 nm light (mauve) and 670 nm light (dark blue). Peak/trough wavelengths are indicated. (*D*) Cph1 was coexpressed with the pre-2 FDBR CAP_1520 (plasmid Spam-545/CAP) and then purified. Absorption spectra are shown for the *15Z* (blue) and *15E* (orange) photostates. (*E*) Normalized photochemical difference spectra are shown as (*15Z*—*15E*) for Cph1 purified after coexpression with pKT271 (PCB control; dark blue line), Spam-545/CAP (cyan line), and Spam-545/8304 (brick red circles). Peak/trough wavelengths are indicated. (*F*) Peak wavelengths for the two bilin transitions in the near-UV to near-IR (300 to 900 nm) are plotted against each other for denatured samples containing BV (filled dark green squares), 18^1^,18^2^–DHBV (open green squares), PΦB (filled dark blue circles), and PCB (open blue circles) adducts and for Cph1 and JSC_58120g3 purified after coexpression with CAP_1520 or pre-3 FDBR MBL9008304 (filled black triangles). For Cph1 with MBL9008304, only the long-wavelength population from the green preparation was plotted. (*G*) Cyanobacterial phytochrome Cph1 was coexpressed with the pre-3 FDBR MBL9008304 (plasmid Spam-545/8304). Cultures giving rise to visibly green cell pellets were pooled, and protein was purified. Absorption spectra are shown using the scheme of panel *D*. (*H*) Cph1 was coexpressed with MBL9008304 as in panel *G*. In this case, cultures giving rise to pink cell pellets were pooled and purified. Absorption spectra are shown using the scheme of panel *D*. (*I*) Peak wavelengths were plotted for denatured samples as in panel *F*. Phycoviolobilin (PVB; filled coral circles), phytoviolobilin (FVB; open mauve circles), and PEB (filled dark blue squares) are compared to the short-wavelength populations of denatured Cph1 (pink preparation; filled black triangle) and BBAGs (open black triangles) after coexpression with MBL9008304.

We first tested the pre-1 protein POZ53557 from *M. psychrotolerans* by replacement of alt-PcyA with POZ53557 in plasmid Spam-545/alt557 (*SI Appendix*, Fig. S6*A*). Purification of Cph1 after coexpression with Spam-545/alt557 gave almost no chromophorylation (Fig. 2*A* and *SI Appendix*, Table S1). Examination of concentrated material revealed two distinct populations (Fig. 2*B*). One population absorbed in the far-red; illumination of this material with far-red light (780 nm) resulted in bleaching without formation of an apparent photoproduct. The other population exhibited red/far-red photoconversion (Fig. 2 *B* and *C*), with peak wavelengths at 672 and 724 nm (*SI Appendix*, Table S1). These peak wavelengths are similar to those reported for Cph1 with a phytochromobilin (PΦB) adduct (36), so POZ53557 appears to carry out inefficient reduction of BV to PΦB. However, poor chromophorylation and low stability of this preparation precluded further characterization.

We next tested the pre-2 protein CAP_1520 from *Chondromyces apiculatus* using plasmid Spam-545/CAP (*SI Appendix*, Fig. S6*A*). Purification of Cph1 after coexpression with Spam-545/CAP gave good yield and chromophorylation (Fig. 2*D* and *SI Appendix*, Table S1). The resulting holo-Cph1 was readily photoconverted between red-absorbing and far-red-absorbing forms (Fig. 2*D*), but its difference spectrum did not match that of Cph1 with a PCB chromophore (Fig. 2*E*). Instead, the observed peak wavelengths (672/722 nm) again matched that of a phytochromobilin (PΦB) adduct [668/718: (36)]. This chromophore assignment was verified using a standard acid denaturation assay (9, 41–45). Denaturation of this preparation (*SI Appendix*, Fig. S7*A*) confirmed the *15E* configuration of the far-red-absorbing form and confirmed that the denatured difference spectrum was red-shifted from that of the bona fide PCB adduct of the CBCR NpR6012g4 [*SI Appendix*, Fig. S7*B*; (46, 47)]. Comparison of the observed *15Z* peak wavelengths to a panel of reference values (Fig. 2*F*) confirmed that this preparation contained a PΦB chromophore. We also tested CAP_1520 with JSC1_58120g3 (9), a CBCR that cannot bind bilins with reduced A-rings such as PCB or PΦB but can bind BV and 18^1^,18^2^–DHBV (*SI Appendix*, Fig. S1 *C* and *D*). We again were able to purify JSC1_58120g3 holoprotein in good yield (*SI Appendix*, Fig. S7*C*). Analysis of this preparation using acid denaturation (*SI Appendix*, Fig. S7*D*) confirmed the expected photoisomerization, and the denatured bilin matched BV (Fig. 2*F* and *SI Appendix*, Fig. S7*E*). Together, these studies show that CAP_1520 is an active FDBR that converts BV only into PΦB.

We next used a similar approach to reconstitute activity for the pre-3 protein MBL9008304. Surprisingly, coexpression of Cph1 with Spam-545/8304 (*SI Appendix*, Fig. S6*A*) gave variable results, with flasks containing green or pink cell pellets at approximately equal frequency. We therefore purified these cultures separately, generating “green” and “pink” preparations. Both MBL9008304-derived preparations exhibited a mixture of two spectrally distinct Cph1 populations with markedly lower chromophorylation than those coexpressed with CAP_1520 (*SI Appendix*, Table S1). Both green and pink preparations possessed photoactive populations, with red- and far-red-absorbing photostates exhibiting absorption maxima matching those obtained with CAP_1520 (674 nm and 710 nm, respectively: Fig. 2 *G* and *H*). A second population with a maximum at 582 nm was present in much greater abundance in the pink preparations (Fig. 2*H*) than that in the green preparations (Fig. 2*G*). This population was photochemically inert and was not seen with CAP_1520 (Fig. 2*D*). Based on the spectral similarity of the photoactive population obtained with both FDBRs (Fig. 2*E*), along with acid denaturation experiments (*SI Appendix*, Fig. S7 *F* and *G*), we assign one of the MBL9008304 products to be the PΦB adduct (Fig. 2*F* and *SI Appendix*, Table S1). The photoinactive pink population was consistent with the known properties of the PEB adduct of Cph1 [Fig. 2*I* and *SI Appendix*, Table S1; (48)]. Coexpression of MBL9008304 with JSC1_58120g3 yielded a product lacking the pink-colored species at 582 nm (*SI Appendix*, Fig. S7*H*), and denaturation experiments revealed BV to be the major chromophore bound (Fig. 2*F*). As JSC1_58120g3 does not bind A-ring-reduced phytobilins, these studies establish that MBL9008304 is an active FDBR which can convert BV to both PΦB and PEB. These studies thus demonstrate that pre-PcyA proteins are active FDBRs able to carry out the same A-ring reduction seen in PcyA.

### Proteins Associated with Pre-PcyA Were Also Acquired by Cyanobacteria.

**Fig. 3.**
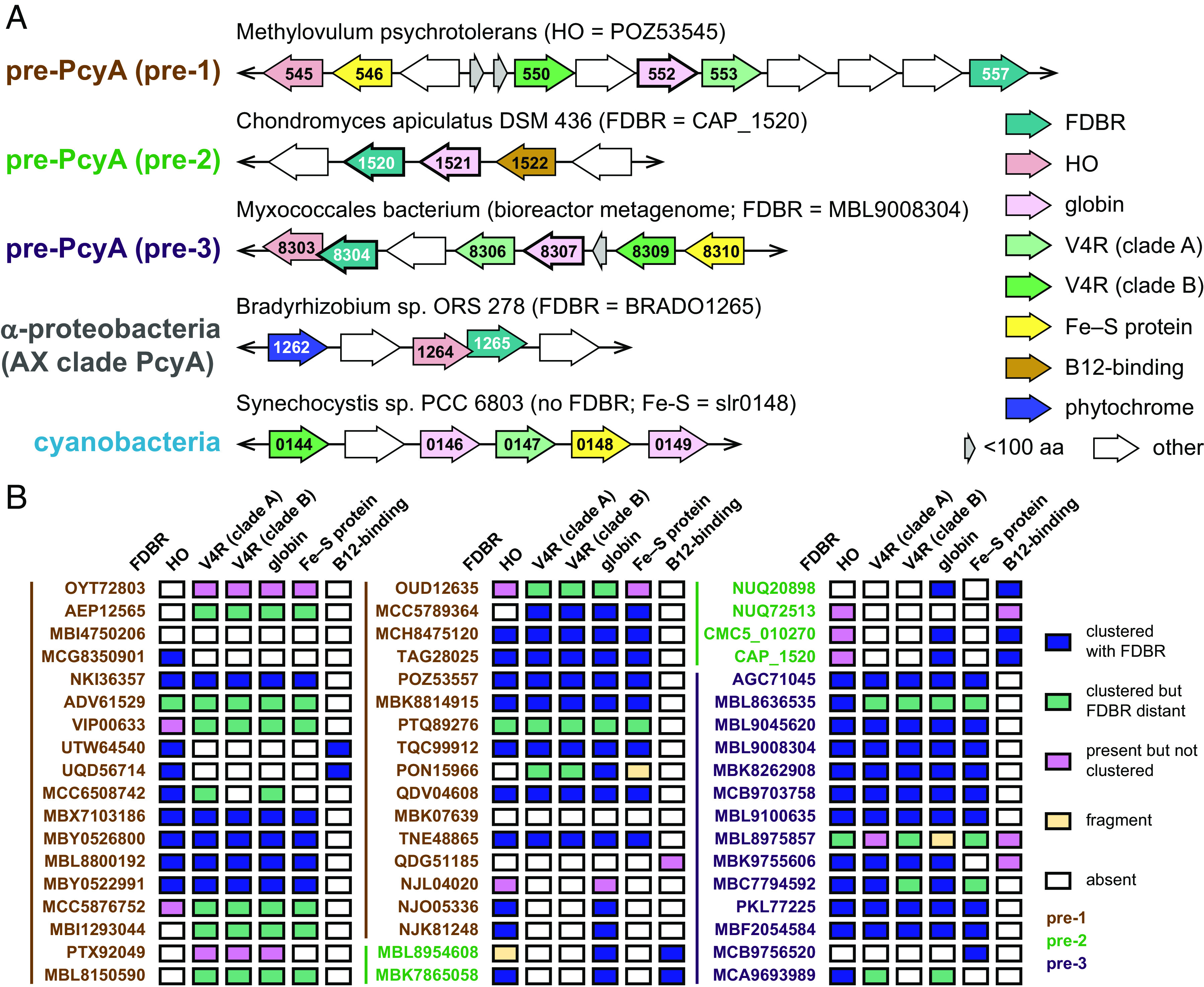
Genomic context of pre-PcyA sequences. (*A*) Open reading frames (ORFs) are shown for pre-PcyA sequences and their neighbors. Representative members of the pre-1, pre-2, and pre-3 lineages are shown, along with a representative AX clade α-proteobacterial PcyA gene from *Bradyrhizobium* sp. ORS 278 and a representative cluster of genes from *Synechocystis* sp. PCC 6803. Conserved genes for each case are color-coded. Overlapping ORFs and direction of the coding strand are indicated, but the figure is not to scale. (*B*) The presence or absence of pre-PcyA-associated proteins from panel A is shown for each pre-PcyA protein included in the phylogenetic analysis of FDBRs (*SI Appendix*, Fig. S3). Gene names are color-coded according to pre-PcyA clade.

We next examined the genomic contexts in which pre-PcyA sequences occur. Previous studies have shown that cyanobacterial *pcyA* genes are found in variable chromosomal contexts, whereas such genes are found in stereotyped loci in the AX clade (11, 12, 22). Pre-1 and pre-3 sequences are found in gene clusters that also typically contain a HO [albeit absent in the anoxygenic photosynthetic genus *Chloracidobacterium* (49)], an iron–sulfur (Fe–S) protein, two proteins with V4R (or 4VR) domains (50, 51), and one or two proteins with homology to phycobiliproteins or globins (representative examples in Fig. 3*A*). Pre-1 clusters are somewhat more variable than pre-3 clusters, with several cases in which one or more genes were distant from the other members of the cluster (Fig. 3*B*). Pre-2 sequences are found in a unique, stereotyped 3-gene cluster also containing a protein with a B12-binding domain and a globin homolog. Neither of these match the context of AX *pcyA* genes (Fig. 3*A*). The pre-1-associated and pre-3-associated clusters also resemble clusters of Fe–S, V4R, and globin genes found in some cyanobacteria, such as the slr0144–slr0149 cluster in *Synechocystis* sp. PCC 6803 (Fig. 3*A*). These genes have been implicated in the assembly and repair of PSI and/or PSII (51–58), although some studies imply that these functions may be mediated by adjacent genes (slr0150–slr0152). Other cyanobacterial examples of this cluster are quite variable: They have been lost in many cyanobacterial genomes but duplicated in others, and the adjacent genes seen in *Synechocystis* are often absent entirely. In the absence of evidence for conservation of these potential functional partners, we focused on the pre-PcyA core cluster genes. We hypothesized that cyanobacterial acquisition of PcyA may have been accompanied by acquisition of other genes in this cluster followed by separation of *pcyA* from the remaining genes in the cluster. We then used a series of phylogenetic analyses (*SI Appendix*, Table S2) to test this hypothesis.

We first examined HO (*SI Appendix*, Fig. S8). Known, structurally homologous HO proteins can exhibit insignificant homology to each other (59), so we did not expect to recover a complete picture of HO evolution. However, several important points were robustly supported by our analysis. First, cyanobacterial HOs are not closely related to the plastid-targeted HO genes of green photosynthetic organisms such as Arabidopsis or Chlamydomonas. Second, glaucophytes and rhodophytes have both cyanobacteria-derived and green-related HOs, with rhodophytes retaining the cyanobacterial HO as a plastid-encoded gene. Third, the HO associated with bacteriophage PebS is closely related to cyanobacterial HOs, whereas that associated with bacteriophage PcyX is distinct both from cyanobacterial HO and from the HO found with α-proteobacterial PcyA (AX clade). A similar discontinuity is found for pre-3 HO enzymes. Pre-3-containing clusters from Myxococcales are associated with a distinct HO, whereas pre-3-associated HOs from Melainabacteria and Sericytochromatia are part of a clade of diverse bacterial HO sequences that also includes those associated with pre-1, pre-2, and AX PcyA (*SI Appendix*, Fig. S8). This analysis thus shows that pre-PcyAs are not associated with an overall dedicated HO lineage and that any pre-PcyA-associated HO potentially transferred to cyanobacteria has since been lost.

**Fig. 4.**
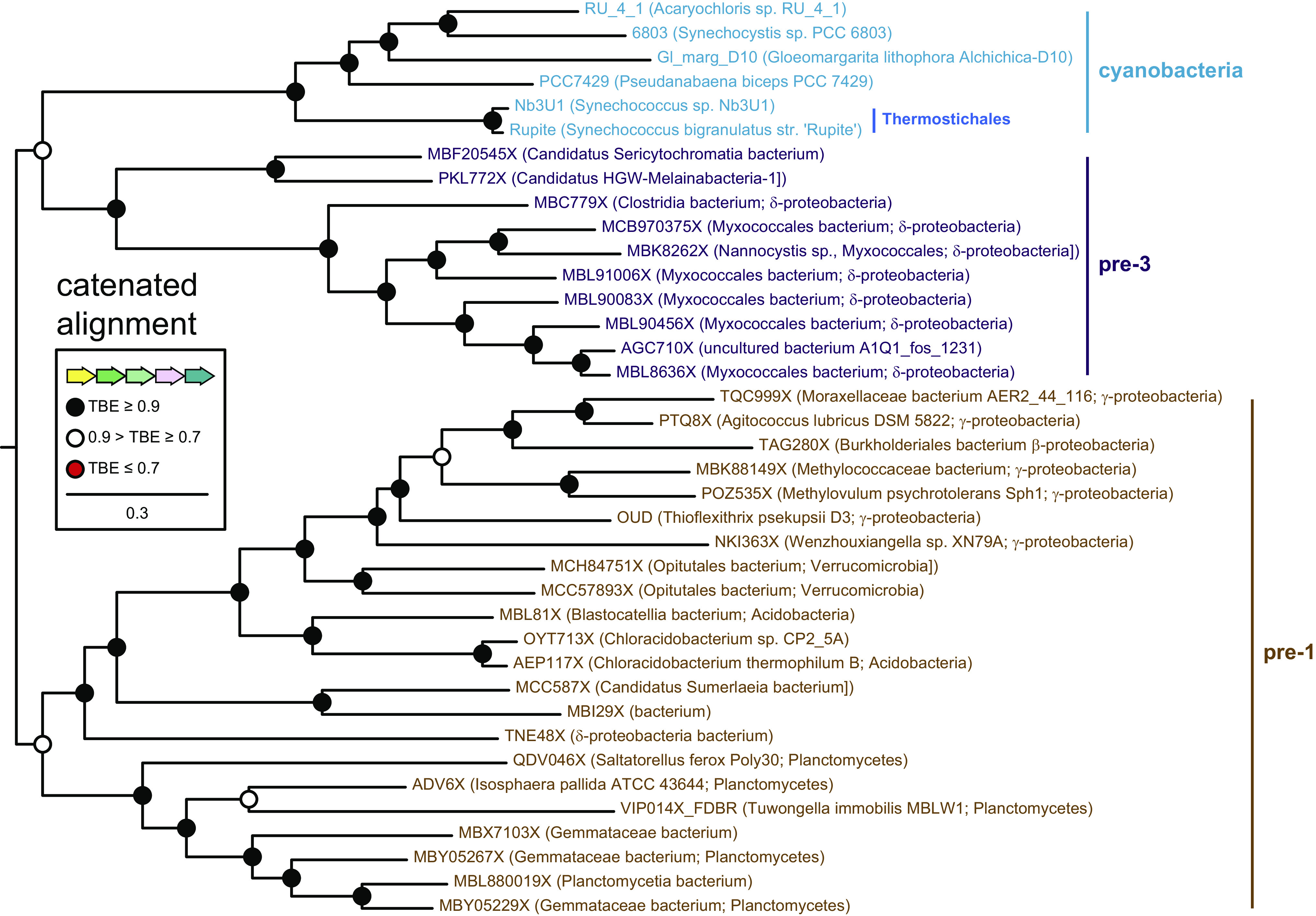
Phylogenetic analysis of proteins associated with pre-1 and pre-3. A catenated alignment comprising the Fe–S protein, V4R pair, globin, and FDBR (Fig. 3*A*) was constructed for pre-1, pre-3, and cyanobacterial proteins. This alignment was used to infer a maximum-likelihood phylogeny as described in Methods and in *SI Appendix*, Table S2. The root placement is equivalent to that of *SI Appendix*, Fig. S3. Support was calculated using the transfer bootstrap expectation value (TBE) as described in the Methods.

By contrast, phylogenetic analysis of Fe–S proteins (*SI Appendix*, Fig. S9) demonstrated that pre-PcyA-associated proteins formed a paraphyletic grade, with cluster-associated cyanobacterial proteins forming a clade derived from the pre-PcyA-associated sequences. Pre-PcyA-associated clusters typically included two V4R proteins (Fig. 3*A*). Phylogenetic analysis demonstrated that those formed two clades, which we designate as clade A and clade B for convenience (*SI Appendix*, Fig. S10). Typical pre-PcyA clusters have one representative of each clade, as do the cyanobacterial gene clusters (Fig. 3*A*). These observations allowed us to carry out a phylogenetic analysis of the FDBRs and associated proteins. To do this, we constructed a catenated alignment comprising Fe–S, V4R, globin, and FDBR proteins. Such catenated analyses can provide a more statistically robust view of the overall evolutionary process; however, relatively few pre-3 clusters had complete sequences for all of the desired proteins. The resulting tree nevertheless provides good support for distinct pre-1, pre-3, and cyanobacterial clades (Fig. 4). If one assumes that the outgroup provided by RCCR in the FDBR tree would apply to this analysis, as shown in Fig. 4, then pre-3-associated clusters and cyanobacterial clusters are sister to each other, and pre-1-associated clusters branched earlier. In other words, the cyanobacterial gene clusters are descended from those associated with pre-PcyA, even though cyanobacterial PcyA is no longer associated with the cluster at the chromosomal level.

### All Pre-PcyA Lineages Are Associated with Globins.

**Fig. 5.**
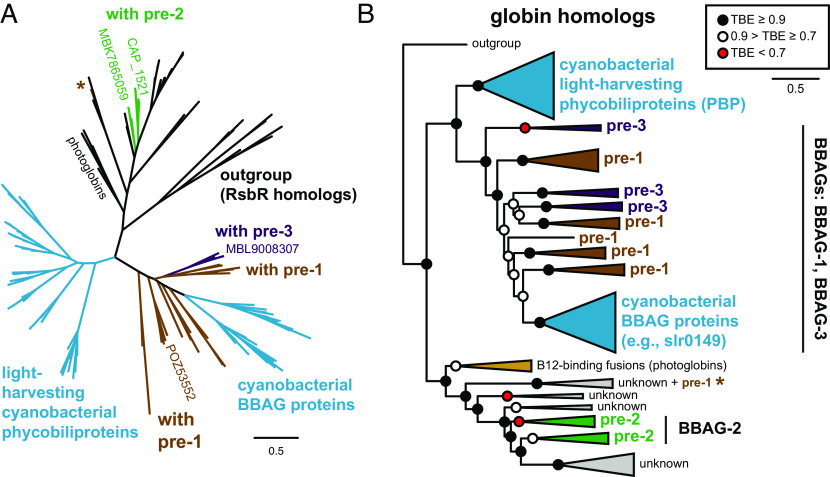
Evolution of bilin-binding globins. A maximum-likelihood phylogenetic tree for globins was inferred as described in *Methods* and in *SI Appendix*, Table S2. The complete tree is presented in unrooted (*A*) and simplified (*B*) views. The position of bilin biosynthesis-associated globins (BBAGs) characterized in this study is shown in panel *A*. For panel *B*, the root is placed between the outgroup and bilin-binding globins as defined in this study. The complete tree is shown in *SI Appendix*, Fig. S11, using the same root placement. Support was calculated using the transfer bootstrap expectation value (TBE) as described in the Methods.

Pre-2 proteins could not be included in the catenated analysis, because they are not found with Fe–S and V4R proteins (Fig. 3*A*). Instead, these proteins are found with a globin and a predicted B12-binding protein. HO sequences are clustered with some pre-2 sequences, but others instead have HO sequences elsewhere in the genome (Fig. 3*B*). As with pre-1, pre-2-associated HO proteins did not form a distinct clade (*SI Appendix*, Fig. S8). We therefore examined globins, the only protein associated with all three pre-PcyA lineages (Fig. 5*A*). Given the general association of these globins with candidate HO and FDBR proteins, we designate them as bilin biosynthesis-associated globins (BBAGs) and number them based on the type of pre-PcyA with which they are associated. For phylogenetic analysis of BBAGs, we also included phycobiliproteins and globin–B12 fusion proteins recently designated as photoglobins (60). Homology to photoglobins allowed us to use the RsbR globin domain (61) as an outgroup. The inferred phylogeny (Fig. 5 and *SI Appendix*, Fig. S11) demonstrates that BBAGs are not a single lineage. Light-harvesting phycobiliproteins form a clade sister to a lineage comprising BBAG-1, BBAG-3, and cluster-associated cyanobacterial globins such as slr0149 (cyanobacterial BBAGs; Fig. 5*B* and *SI Appendix*, Fig. S11). Consistent with the catenated analysis (Fig. 4), this result demonstrates that cyanobacterial BBAGs were acquired along with other genes associated with pre-1 and pre-3 and are distinct from light-harvesting phycobiliproteins.

Pre-2-associated globins (BBAG-2) belong to a distinct lineage that is sister to the combined clade comprising BBAG-1, BBAG-3, cyanobacterial BBAGs, and phycobiliproteins (Fig. 5*B*). The BBAG-2 lineage also includes photoglobins (60) and globins associated with the atypical pre-1 sequences NJL04020 and NJO05336 (Fig. 3*B*). Unlike photoglobins, BBAG-2 proteins do not form a distinct clade within this lineage (*SI Appendix*, Fig. S11). Instead, BBAG-2 belongs to a clade also including globins from a range of Myxococcales. Phylogenetic analysis of the B12-binding proteins associated with pre-2 revealed a similar pattern (*SI Appendix*, Fig. S12). Moreover, the Myxococcales BBAG sequences not associated with pre-2 were still associated with B12-binding proteins and were found in genomes also encoding HO proteins (*SI Appendix*, Figs. S11 and S12). BBAG-2 would therefore seem to be a special case of a globin generally associated with HO and B12-binding proteins. HO converts heme into biliverdin (*SI Appendix*, Fig. S1*A*), so such proteins are also associated with bilin biosynthesis. This analysis thus demonstrates that BBAGs associated with different pre-PcyA lineages are part of a larger clade also including photoglobins and phycobiliproteins.

### BBAGs Are Bilin-Binding Proteins.

To assess potential bilin binding in BBAGs, we examined potential sites of chromophore attachment. Phycobiliproteins frequently use a CXRD motif for chromophore binding, with the Cys residue forming a covalent attachment to the bilin A-ring and the other two conserved residues providing hydrogen bonding partners to other parts of the chromophore (62, 63). Some BBAG-1, BBAG-3, and cyanobacterial BBAGs also have this motif, whereas photoglobins have a related CXQD motif (*SI Appendix*, Fig. S11). A distinct Cys-containing RGMC motif was found in BBAG-2 (*SI Appendix*, Fig. S11). This motif was also found in some of the Myxococcales globins also associated with B12-binding proteins and with HO, but not with pre-2 (*SI Appendix*, Figs. S11 and S12). We therefore sought to assess potential bilin binding by BBAGs using the reconstitution approach that successfully verified FDBR function.

**Fig. 6.**
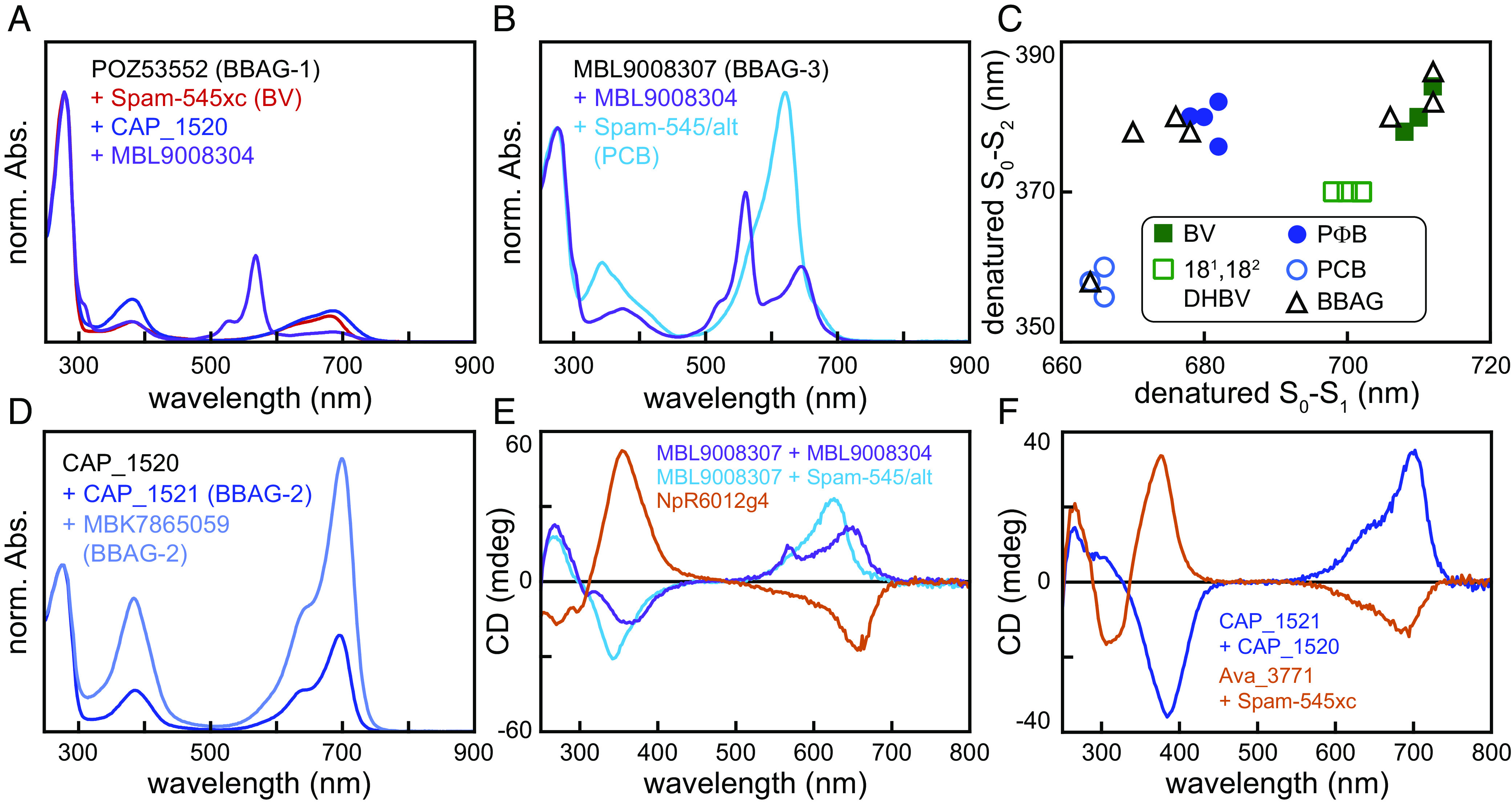
Characterization of bilin biosynthesis-associated globins (BBAGs). Candidate BBAGs were expressed recombinantly in *E. coli* also expressing different bilin biosynthesis plasmids. (*A*) BBAG-1 POZ53552 was coexpressed with the HO POZ53545 with no FDBR (synthesizing BV; plasmid Spam-545xc, *SI Appendix*, Fig. S6*A*), POZ53545 with FDBR CAP_1520 (plasmid Spam-545/CAP), and POZ53545 with FDBR MBL9008304 (plasmid Spam-545/8304). Normalized absorption spectra are shown for purified protein (POZ53545 alone, red; CAP_1520, dark blue; MBL9008304, purple). (*B*) BBAG-3 MBL9008307 was coexpressed with its cognate pre-3 FDBR MBL9008304 (Fig. 3*A*) or with POZ53545 and PcyA (plasmid Spam-545/alt). Normalized absorption spectra are shown for purified protein (MBL9008304, purple; PcyA, cyan). (*C*) Peak wavelengths are plotted for denatured samples of BBAGs (open black triangles) and reference bilin adducts as in Fig. 2*F*. (*D*) Normalized absorption spectra are shown for purified BBAG-2 CAP_1521 (dark blue) and BBAG-2 MBK7865059 (periwinkle) after coexpression with CAP_1520. (*E*) Circular dichroism (CD) spectra are shown for the samples in panel *B* and for the CBCR NpR6012g4 (burnt sienna) over the near-UV/visible region matching the absorption spectra. (*F*) CD spectra are shown for the samples in panel *D* and for CBCR Ava_3771 incorporating BV (burnt sienna; *SI Appendix*, Fig. S6*C*). All normalized spectra were normalized on the aromatic amino acid band at ~280 nm.

We first examined POZ53552, a BBAG-1 associated with the HO POZ53545 and the pre-1 FDBR POZ53557 (Fig. 3*A*). Coexpression of POZ53552 was carried out either with POZ53545 HO alone (plasmid Spam-545xc, *SI Appendix*, Fig. S6*A*) or together with the FDBRs POZ53557 (Spam-545/alt557), CAP_1520 (Spam-545/CAP), or MBL9008304 (Spam-545/8304). In each case, we obtained a poorly chromophorylated holoprotein that was photoinactive (Fig. 6*A* and *SI Appendix*, Table S1). The presence or absence of POZ53557 did not change the outcome relative to the HO alone (*SI Appendix*, Table S1), consistent with its poor FDBR activity (see above). We next examined MBL9008307, the cognate BBAG-3 for MBL9008304 (Fig. 3*A*). Chromophorylation of BBAG-3 MBL9008307 was notably better than that of BBAG-1 POZ53552 with either *Synechocystis* PcyA, using plasmid Spam-545/alt, or with its cognate FDBR MBL9008304, using plasmid Spam-545/8304 (Fig. 6 *A* and *B* and *SI Appendix*, Table S1). For both BBAGs, we observed multiple peaks with MBL9008304 (Fig. 6 *A* and *B* and *SI Appendix*, Table S1), consistent with the multiple bilin adducts observed for Cph1 with this FDBR (Fig. 2 *G* and *H*). Denaturation analysis confirmed that these peaks reflected a mixture of PΦB and PEB (Figs. 2*I* and 6*C*). As seen with Cph1 as a reporter, BBAG-1 POZ53552 incorporated PΦB when coexpressed with Spam-545/CAP, and BBAG-3 MBL9008307 incorporated PCB when coexpressed with PcyA (Fig. 6*C* and *SI Appendix*, Table S1). We next examined two BBAG-2 proteins: CAP_1521 is the cognate globin for the pre-2 FDBR CAP_1520 (Fig. 3*A*), and MBK7865059 is associated with the uncharacterized pre-2 FDBR MBK7865058 (*SI Appendix*, Fig. S3). Both proteins were coexpressed with Spam-545/CAP in *E. coli*. BBAG-2 CAP_1521 exhibited modest chromophorylation, whereas BBAG-2 MBK7865059 exhibited excellent chromophorylation (Fig. 6*D*). However, both holoproteins were photoinert. Acid denaturation demonstrated that both possessed BV adducts rather than PΦB adducts (Fig. 6*C* and *SI Appendix*, Fig. S13 *A* and *B*), a surprising result in light of the facile reconstitution of Cph1 with PΦB produced by CAP_1520 (Fig. 2*D*). Taken together, these results establish that all the three pre-PcyA lineages are associated with authentic bilin-binding globins.

To further characterize BBAGs, we used fluorescence spectroscopy. Light harvesting by phycobiliproteins relies on ET between precisely positioned, photochemically correct bilin chromophores within a large phycobilisome complex (10, 64, 65), but isolated phycobiliproteins exhibit very high fluorescence in the absence of suitable acceptors for ET. For example, reported quantum yields for the light-harvesting phycobiliprotein C-phycocyanin are species dependent but can be as high as 80% (66). We first examined MBL9008307 with PCB chromophore. Fluorescence excitation and emission were readily detected (*SI Appendix*, Fig. S13*C*), with comparison of the absorption and excitation spectra indicating possible heterogeneity. We used the ratio method (37, 39) with the commercially available dye Alexa647 to estimate the quantum yield of MBL9008307 at 3% with PCB chromophore (*SI Appendix*, Fig. S13*D*). We next examined the BBAG-2 proteins CAP_1521 and MBK7865059, both with BV chromophore. We were able to measure excitation and emission spectra for both proteins (*SI Appendix*, Fig. S13 *E* and *F*), again observing spectral heterogeneity. We used Alexa700 to estimate quantum yields for these proteins (*SI Appendix*, Fig. S13*G*), obtaining slightly higher values of 4% for MBK7865059 and 7% for CAP_1521. BBAGs thus do not exhibit high intrinsic fluorescence, in contrast to light-harvesting phycobiliproteins.

Finally, we also characterized BBAGs using circular dichroism (CD) spectroscopy. Secondary structure was examined for MBL9008307 incorporating PCB and MBK7865059 incorporating BV, the samples with the highest chromophorylation. Both proteins exhibited CD spectra consistent with folded, primarily α-helical structures (*SI Appendix*, Fig. S13*H*). Since bilins bound to proteins typically adopt asymmetric configurations in three-dimensional space and therefore are CD active (67, 68), we also examined the bilin transitions of BBAGs by CD. Isolated phycobiliprotein monomers typically exhibit positive CD on the low-energy transition (the S_0_–S_1_ transition, such as the red-absorbing band of phycocyanin) and negative CD for the near-UV transition [S_0_–S_2_ or Soret band: (42, 69)]. By contrast, the *15Z* configuration of phytochromes and CBCRs exhibits the opposite CD (45, 67, 68, 70, 71). Consistent with these assignments, the model CBCR NpR6012g4 and BBAG MBL9008307 exhibited opposite CD when incorporating the same PCB chromophore (Fig. 6*E*). MBL9008307 coexpressed with its cognate pre-3 allowed us to examine PΦB and PEB populations, both of which exhibited the same CD signature as the PCB adduct. Similarly, BBAG CAP_1521 and CBCR Ava_3771 exhibited opposite CD with BV chromophore: Ava_3771 matched NpR6012g4 and CAP_1521 matched MBL9008307 (Fig. 6*F*). The CD spectrum of MBK7865059 also matched those of the other BBAGs (*SI Appendix*, Fig. S13*I*). Despite the distant relationships between BBAG-2, BBAG-3, and phycobiliproteins, we conclude that these three bilin-binding globins all exhibit a conserved CD signature distinct from that of 15Z phytochromes and CBCRs.

## Discussion

In this work, we provide insight into the origins of PCB biosynthesis and bilin-based light harvesting in cyanobacteria. Our studies examine the interconnected evolutionary histories of PcyA and of bilin-binding globins and have important implications regarding cyanobacterial acquisition and evolution of PcyA activity as well as the evolution of phycobiliproteins.

### Acquisition of PcyA by Cyanobacteria.

Our phylogenetic analysis (Fig. 1 and *SI Appendix*, Figs. S2 and S3) demonstrates that PcyA arose from pre-PcyA FDBRs found in heterotrophic bacteria. Using one example from each of the pre-PcyA lineages to reconstitute activity in *E. coli,* we also establish that pre-PcyA proteins are active FDBRs that convert BV into phytobilins. The pre-1 enzyme examined in this study was apparently able to generate small amounts of PΦB. This result suggests that either the enzyme is poorly active or that POZ53557 yields a mixture of reduced bilin products, comprised of PΦB and possibly others that are unable to support photoactive adducts with Cph1. By comparison, both pre-2 and pre-3 enzymes exhibited robust 2-electron reduction of BV into PΦB in this assay. The pre-3 FDBR MLB9008304 also carries out a 4-electron reduction to produce PEB. Reduction of the BV into PΦB proceeds via reduction of the BV A-ring (*SI Appendix*, Fig. S1), which also occurs during synthesis of PCB by PcyA. PEB formation by pre-3 apparently proceeds via initial PΦB formation, in contrast to the initial formation of 15,16-dihydrobiliverdin in previously described pathways [*SI Appendix*, Fig. S1 *C* and *D*; (12, 17, 22, 72)]. These results demonstrate that the regiospecificity of pre-PcyA enzymes has some overlap with that of PcyA itself. From these active pre-PcyA proteins, evolution of PCB biosynthesis apparently proceeded via addition of Glu76 to the active site. This residue is specific for PcyA enzymes (*SI Appendix*, Fig. S3) and is required for the characteristic PcyA reduction of the BV 18-vinyl moiety [*SI Appendix*, Fig. S1 *C* and *D*; (31)]. Within the PcyA lineage, the early-branching AX clade includes PcyA from α-proteobacteria, whereas most pre-2 and pre-3 sequences are found in δ-proteobacteria. AX PcyA is known to be active and to synthesize PCB (11, 12), unlike pre-PcyA. We therefore propose that evolution of PcyA occurred within the proteobacteria via acquisition of Glu76 and 18-vinyl reduction regiospecificity.

Pre-1 and pre-3 are associated with a cluster of characteristic proteins in bacterial chromosomes and MAGs, comprising an Fe–S protein, a BBAG, two V4R proteins, and sometimes an HO (Fig. 3). Similar clusters are also found in cyanobacteria but lack the FDBR and HO. Phylogenetic analysis demonstrates that pre-PcyA proteins are not associated with a discrete HO clade (*SI Appendix*, Fig. S8), in contrast to the other proteins in this cluster (*SI Appendix*, Figs. S9–S11). Moreover, phylogenetic analysis of a catenated alignment of cluster proteins demonstrated that they followed a similar evolutionary pattern to that of FDBRs alone, with cyanobacterial proteins and pre-3 proteins as sister lineages and pre-1 proteins as an earlier branch. Evolution of PcyA also follows the pattern expected for an ancestral cyanobacterial gene, with sequences from the early-branching Gloeobacterales and Thermostichales (23, 24, 73) branching in the correct order before sequences from more derived cyanobacteria. Cyanobacterial cluster genes are absent in available Gloeobacterales genomes and MAGs, but those from members of the Thermostichales are again basal relative to other cyanobacterial sequences (Fig. 4). We therefore propose that a common ancestor of extant cyanobacteria acquired PcyA via horizontal gene transfer of a larger segment from a proteobacterium.

### Acquisition of Bilin Binding by Globins.

All of the three pre-PcyA lineages are associated with globins, which we designate as BBAGs. BBAGs associated with pre-1 and pre-3 (BBAG-1 and BBAG-3, respectively) are part of a single lineage, and cyanobacterial BBAGs form a clade descended from these sequences (*SI Appendix*, Fig. S11). Representative BBAG-1 and BBAG-3 proteins are able to bind a range of bilins in the absence of bilin lyases. This situation is somewhat reminiscent of phycobiliproteins, which typically do require lyases but are also able to incorporate a range of bilins (74, 75). Moreover, many phycobiliproteins contain a CXRD motif including the Cys residue required for covalent chromophore attachment. The same motif is present in the BBAG-1 and BBAG-3 proteins examined in this study (*SI Appendix*, Fig. S11). Light-harvesting phycobiliproteins are sister to the BBAG-1/BBAG-3 lineage, so together they form a clade of bilin-binding globins sister to the clade including photoglobins and BBAG-2. It is clear from phylogenetic analysis that phycobiliproteins were not acquired by cyanobacteria along with PcyA and cyanobacterial BBAGs (*SI Appendix*, Fig. S11), so the precise origin of phycobiliproteins remains unclear. However, our analysis demonstrates that BBAG-2 proteins are also bilin binding. These proteins lack the CXRD motif and have distinct bilin specificity: Unlike BBAG-1 and BBAG-3, BBAG-2 is specific for biliverdin (Fig. 6). We therefore conclude that phycobiliproteins evolved within a larger, preexisting clade of bilin-binding globins which were adapted for light harvesting by cyanobacteria.

The ancestral bilin-binding globins probably bound BV, which requires only HO for synthesis from heme (*SI Appendix*, Fig. S1*A*), and BBAG-2 retains specificity for this ligand. CD spectroscopy demonstrates that the overall conformation of bilin chromophores in BBAGs adopts a similar position to that of phycobiliproteins, but fluorescence spectroscopy demonstrates that BBAGs do not have the high fluorescence characteristic of isolated phycobiliproteins (*SI Appendix*, Fig. S13). Current data thus indicate that extant BBAGs are not efficient light-harvesting proteins, and their functions are not yet known. Diversification of ancestral BBAGs gave rise to a common ancestor of phycobiliproteins and BBAG-1/BBAG-3 proteins which may have been able to bind a wider range of bilins using the CXRD motif found in modern phycobiliproteins. BBAG-1 remained clustered with the coevolving FDBR to give rise to BBAG-3, and an HGT event gave rise to cyanobacterial PcyA and cyanobacterial BBAGs but not phycobiliproteins.

The emerging importance of phycobiliproteins in cyanobacterial light harvesting would have provided a selective advantage for PcyA to be regulated independently of BBAGs and other cluster-associated proteins. The function of cyanobacterial BBAGs and associated proteins is not essential and may not be conserved, because such proteins are absent in a broad range of cyanobacteria including the early-branching genus *Gloeobacter*, the developmentally sophisticated filamentous cyanobacterium *Nostoc punctiforme,* and the abundant marine picocyanobacterium *Prochlorococcus.* There are also cases of BBAG duplication within a cluster and of full or partial cluster duplication, both in cyanobacteria and in pre-1 clusters. By contrast to the uncertain functions of BBAGs, phycobiliproteins have a well-established function in light harvesting via ET, requiring them to assemble into a large array with chemically defined, precisely positioned bilin chromophores. In assembling this phycobilisome antenna, the dependence of phycobiliproteins on lyases for efficient chromophorylation would be expected to provide a functional advantage: Lyases provide specific incorporation of a given, well-defined bilin at a specific site which then adopts a specific position within the larger phycobilisome complex (74, 76). It is also possible that lyases may aid in reducing heterogeneity, which is implicated in BBAGs by fluorescence spectroscopy and is well established in other biliproteins (47, 77, 78). We therefore propose that specific adaptations of phycobiliproteins for light harvesting include loss of efficient self-assembly with bilins and gain of high intrinsic fluorescence.

### Bilin Biosynthesis and the Rise of Oxygenic Photosynthesis.

Our studies also further speculation about the evolutionary and ecological processes involved in assembling the modern cyanobacterial photosynthetic apparatus. There are implicit contradictions in these processes: HO requires oxygen, but cyanobacterial oxygenic photosynthesis relies on bilin chromophores whose synthesis requires HO. However, even low levels of oxygen can be toxic in the presence of light and tetrapyrroles. Oxygen can also impact heme synthesis pathways (79). The anaerobic pathway for heme synthesis also contains enzymes that are inactivated by oxygen, such as the oxygen-independent coproporphyrinogen III oxidase HemN (80). HemN is a radical SAM enzyme containing an Fe–S cluster and is structurally unrelated to its oxygen-dependent analog, HemF or coproporphyrinogen oxidase (CPOX) (81). This situation is also reminiscent of differences between anaerobic and aerobic pathways for heme breakdown: One anaerobic pathway begins with the radical SAM enzyme ChuW and proceeds via the reductase ChuY (82–84), whereas one aerobic pathway begins with HO and proceeds via pre-PcyA or PcyA. Interestingly, there is another link between heme synthesis and heme turnover. CPOX adopts the same mixed α/β fold as FDBRs and RCCR, even though the amino acid similarity of CPOX to these proteins is not statistically significant (85). Both RCCR and FDBRs act subsequent to oxygen-dependent enzymes [HO and pheophorbide a oxygenase, respectively; (86)], whereas CPOX is directly dependent on molecular oxygen (87, 88). Hence, the known enzymes structurally related to pre-PcyA and PcyA are all ultimately dependent on the presence of oxygen for function. We therefore propose that FDBRs arose after initial evolution of oxygenic photosynthesis alongside evolution of RCCRs and CPOX, perhaps reflecting a larger remodeling of tetrapyrrole metabolism to provide functional pathways in the presence of oxygen.

However, our work provides evidence that PcyA did not evolve in an oxygenic photosynthetic organism. It is perhaps noteworthy that pre-2 and pre-3 FDBRs are primarily found in Myxococcales, which are complex, social heterotrophs belonging to the δ-proteobacteria. We thus can envision early cyanobacteria growing in a microbial community alongside such organisms. As nascent photosynthesis began to produce small amounts of oxygen in light, development of bacterial HOs would have provided a means of detoxification that elegantly eliminated both oxygen and free heme in a single reaction. These ancestral HO proteins could then have provided BV to an ancestral bilin-binding globin. This protein could have provided further protection against reactive oxygen species by analogy to the photoprotective role played by bilin and GUN4 in *Chlamydomonas* today (6). We therefore envision that cyanobacterial bilin biosynthesis pathways could have begun as detoxification pathways in neighboring bacteria, with the evolution of efficient oxygenic photosynthesis leveraging innovations not only in early cyanobacteria but also in other members of the microbial community in which they lived.

## Materials and Methods

### Phylogenetic Analysis and Homology Modeling.

BLAST (89) was used to search for sequences for analysis using a range of queries. In addition to the NCBI database, we also used bacterial genomes and MAGs downloaded from NCBI, eukaryotic transcriptomes from NCBI and from the Marine Microbial Eukaryote Transcriptome Sequencing Project and 1KP datasets (90, 91), and additional datasets (92–96). NCBI searches were sometimes taxonomically constrained (for example by suppressing cyanobacterial sequences) to facilitate detection of distant homologs. Multiple sequence alignments were constructed in MAFFT v7.450 (97). For maximum-likelihood phylogenetic analysis, the resulting alignment was processed with an in-house script to remove positions having ≥5% gaps. Phylogenies were inferred using PhyML-3.1 (98) with 100 bootstraps. Statistical robustness was assessed using the transfer bootstrap expectation [TBE, (99)] as implemented in booster (99). Trees were analyzed using FigTree v1.4.4. Command-line settings and additional information are presented in *SI Appendix*.

To assess candidate active-site residues in pre-PcyA proteins, homology models were prepared for POZ53557, CAP_1520, and MBL9008304 in MODELLER version 9.22 (100) using *Synechocystis* PcyA with bound BV [PDB accession 2D1E; (85)] as a template and using the multiple sequence alignment also used for phylogenetic analysis, but with gap-enriched positions retained. Sequences of interest were extracted from the sequence alignment without changing the aligned positions using the alnfilter utility [available as part of the homolmapper distribution; (101)] and were converted to PIR format using the -convert command-line option in CLUSTAL (102). The resulting .pir files were edited manually to generate inputs for MODELLER, and the resulting models are presented in *SI Appendix*, Fig. S4. As an independent test, predicted structures for PTQ89276 from *Agitococcus lubricus* (pre-1), CAP_1520, and PKL77225 (pre-3) generated by AlphaFold (33) were downloaded from the online database (alphafold.ebi.ac.uk) and superimposed onto the PcyA structure (*SI Appendix*, Fig. S5). Structural figures were prepared using VMD and Tachyon (103, 104). The C-terminal region of CAP_1520 (amino acids 240 to 283) was not folded in the predicted structure from AlphaFold and was omitted from *SI Appendix*, Fig. S5 for clarity.

### Plasmid Construction.

For reconstitution of PCB biosynthesis, we used plasmid Spam-545/PcyA as a starting point. Spam-545/PcyA was constructed from pKT271 (35, 39) by introducing additional BamHI and SpeI restriction sites to generate Spam-pKT (*SI Appendix*, Fig. S6*A*). Based on the TIR-2 sequence recently reported to improve protein expression in the pET28 family (38), we designed an alt-PcyA construct (alt-PcyA, *SI Appendix*, Fig. S6*B*). This was cloned into Spam-pKT or Spam-545/PcyA using BamHI and SpeI to generate Spam-ho1/alt and Spam-545/alt, respectively (*SI Appendix*, Fig. S6*A*). These constructs were evaluated with the previously published expression construct for the CBCR domain of Ava_3771 (*SI Appendix*, Fig. S6 *C*–*E* and Table S1), originally characterized using pKT271 (40). Plasmid Spam-545/alt was chosen as the best scaffold for FDBR reconstitution, so synthetic genes having the alt-PcyA 5′ end but encoding CAP_1520 or MBL9008304 (GenScript) after the linker were cloned into the same BamHI and SpeI sites to generate Spam-545/CAP and Spam-545/8304 (*SI Appendix*, Fig. S6*A*). Additional details are presented in Supplementary Information and in *SI Appendix*, Table S1.

### Protein Expression and Purification.

Expression and purification of His-tagged Ava_3771 (40) and His-tagged Cph1 [using the N514 truncation construct sufficient for photoconversion; (14)] followed previously published procedures except that bilin biosynthesis was supplied with a range of plasmids carrying different HO and FDBR sequences. Expression and purification of His-tagged JSC1_58120g3 followed the same procedures, using pET28-JSC1_58120g3 (generous gift of Marcus V. Moreno). For expression of BBAGs, synthetic genes were acquired as synthetic genes from Genscript and were cloned into pET28-RcaE (105). Expression and purification of BBAGs was then carried out as for phytochromes or CBCRs. JSC1_58120g3 containing BV and NpR6012g4 containing PCB were used as reference samples with known chromophore structure (9, 47). These samples were purified using the previously described intein–CBD fusion constructs, except that NpR6012g4 growth was performed entirely in LB relative to the normal procedure using RM media (34, 69).

### Spectroscopy.

Absorption spectra were acquired using Cary50 or Cary60 spectrophotometers at 25 °C (250 to 900 nm, sampled every 2 nm for 0.125 s), and CD spectra were acquired using a Chirascan CD spectrometer (Applied Photophysics). Photoconversion of Cph1 and of CBCRs was triggered using red and green laser pointers, a xenon lamp fit with a 670 nm bandpass filter, and far-red LEDs (728 nm; Sanyo; 780 nm, ThorLabs). BBAGs were similarly illuminated, but photoconversion was not observed. For acid denaturation, 100 µL protein samples were diluted in 1 mL 7M guanidinium chloride/1% HCl (v/v). Absorption spectra were then recorded as for native samples, and photoconversion was triggered using top-down illumination with the xenon lamp fit with a 320-nm long-pass filter (106). In this assay, the 15E isomer is much more photoreactive than the 15Z isomer (41–43, 45). allowing assignment of the bilin configuration. Assignment of the bilin identity used the denatured 15Z spectrum (to avoid artifacts associated with incomplete photoconversion), the denatured photochemical difference spectrum (to assess photoactive populations when present), and plotting peak wavelengths for the two bilin transitions versus reference samples with known bilin content. To examine CD signatures from bilins, spectra were acquired over the near-UV/visible window (250 to 850 nm, sampled every 1 nm) using the same samples as for native absorption spectra. To examine secondary structure, 50 µL sample was added to 3 mL HPLC-grade water, and an absorption spectrum was taken in a 1 cm square cuvette (190 to 900 nm; see above). A volume of 300 µL was then transferred to a fixed-mount cuvette with 1 mm path length, and duplicate CD spectra were acquired. All spectra were baseline subtracted. Duplicate CD spectra were averaged. Fluorescence spectra were acquired on a QM-6/2005SE fluorimeter equipped with red-enhanced photomultiplier tubes (Photon Technology International 814 Series). Fluorescence quantum yields were estimated using a ratio approach (37, 39) with Alexa647 or Alexa700 dyes (ThermoFisher) as references with known quantum yields. Dilution series were prepared for reference and unknown samples. Absorbance and emission spectra were taken for each sample and concentration, using a constant excitation wavelength for emission spectra. Numerically integrated emission was plotted versus absorbance at the excitation wavelength and fit by linear regression. In this analysis, the fitted slope is proportional to the quantum yield, allowing the fluorescence quantum yield of the unknown sample to be estimated from the ratio of its slope to the slope of the reference. Regression analyses were performed in Kaleidagraph.

## Supplementary Material

Appendix 01 (PDF)

## Data Availability

Raw spectra, multiple sequence alignments before and after gap removal, resulting tree files, homology models, and synthetic gene sequences are available via DataDryad (107) along with the full plasmid sequence for Spam-545/alt.
