# Supplementary material for: Elucidating the origins of phycocyanobilin biosynthesis and phycobiliproteins
Source: Proc Natl Acad Sci U S A. 2023 Apr 18;120(17):e2300770120. doi: 10.1073/pnas.2300770120 (PMC10151467; doi:10.1073/pnas.2300770120)
Supplement: Supplementary file 1 — Appendix 01 (PDF) [file pnas.2300770120.sapp.pdf]

PNAS

Supplementary Information for

Elucidating the origins of phycocyanobilin biosynthesis and phycobiliproteins

Nathan C. Rockwell*, Shelley S. Martin, and J. Clark Lagarias*

Nathan C. Rockwell
Email: ncrockwell@ucdavis.edu

J. Clark Lagarias
Email: jclagarias@ucdavis.edu

This PDF file includes:

Supplementary text
Figures S1 to S13
Tables S1 to S2
SI References

Supplementary Information Text

Methods.

Phylogenetic analysis. Multiple sequence alignments were constructed in MAFFT v7.450 using the command-line settings --genafpair --maxiterate 16 --clustalout –reorder. For maximum-likelihood phylogenetic analysis, the resulting alignment was processed with an in-house script to remove positions having ≥5% gaps. Phylogenies were inferred with PhyML-3.1 with 100 bootstraps, using the command-line settings -m WAG -d aa -s SPR -a e -c 4 -v e -o tlr -b 100. Statistical robustness was assessed using the transfer bootstrap expectation (TBE) as implemented in booster (1). Completeness of sequences after gap removal was assessed using the —length option of alnfilter, a utility available as part of the homolmapper distribution (2). The number of sequences and characters (after gap removal) used for inferring each phylogeny is presented in Table S2, along with the number of sequences <90% complete after gap removal. For FDBRs, a branch containing RCCR was considered to be the outgroup. For HO, we were unable to find a clear, suitable outgroup; therefore, root placement was arbitrary. For Fe–S proteins, the outgroup included putidaredoxin and related bacterial Fe–S proteins. For V4R proteins, the outgroup included bacterial and archaeal V4R sequences not associated with pre-PcyA. For globins, the outgroup included rsbR globin domains and related sequences. The catenated alignment did not include an outgroup; root placement was based on the phylogenetic analysis of FDBRs.

Plasmid design. Spam-545/PcyA was the starting point for reconstitution of pre-PcyA activity and was constructed from pKT271 (step 1, Fig. S3A) via introduction of additional restriction sites (3). The open reading frame for the heme oxygenase gene from *Synechocystis* sp. PCC6803 was then replaced with that for heme oxygenase POZ53545 using unique NdeI and XhoI sites (step 2). This retained the sequence encoding *Synechocystis* PcyA; excision of that by cleavage with SalI and XhoI and ligation of the compatible sticky ends gave rise to plasmid Spam-545xc (step 3), which exhibited higher chromophorylation than an equivalent excision of Spam-pKT (4). In the current work, we sought to improve PcyA expression and/or function in this context. In pKT271, Spam-545/PcyA, and related plasmids, the region upstream of the PcyA open reading frame lacks a canonical Shine-Dalgarno sequence (Fig. S3B). However, the introduced BamHI site immediately 5' to the ATG codon also introduces additional homology to the recently described pET28 derivative TIR-2 (5). TIR-2 improves expression via changes both in this region and in the second and third codons of the expressed sequence. We therefore designed an alternative 5' end for the PcyA open reading frame incorporating these codons along with a FLAG tag and a short linker peptide (Fig. S3B). This alt-PcyA construct was then cloned into Spam-pKT and Spam-545/PcyA to yield Spam-ho1/alt and Spam-545/alt (steps 4 & 5, Fig. S3A). All four plasmids were tested by co-expression with Ava_3771 (6), with Spam-545/alt giving the best chromophorylation and PCB incorporation (Fig. S3C-E & Table S1). This plasmid was then used as a scaffold for testing other FDBRs (step 6, Fig. S3A), using the "alt" 5-prime end and N-terminal sequence. All constructs were verified by nucleotide sequencing.

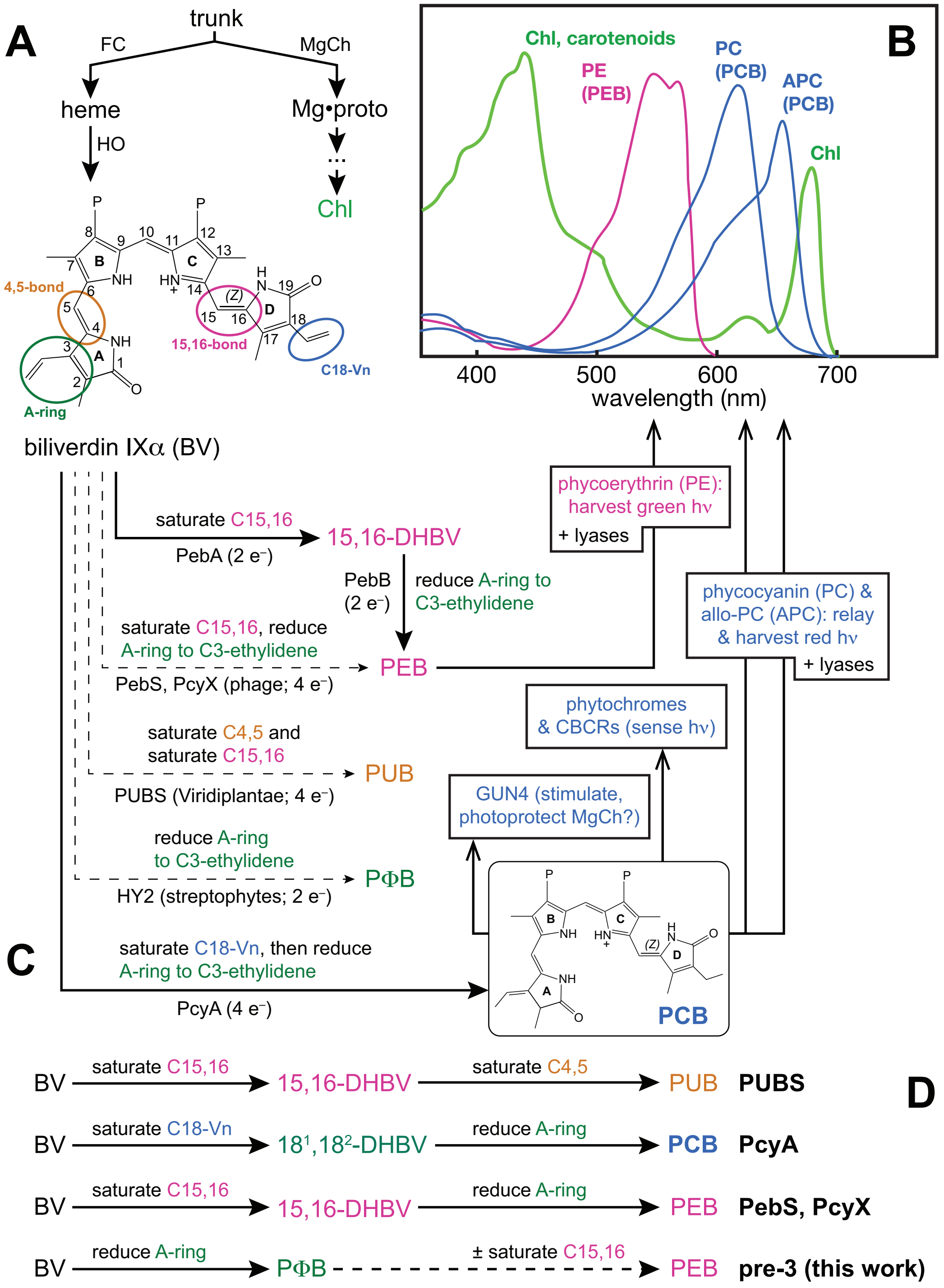

Fig. S1. Bilin biosynthesis and function in cyanobacteria. (A) Synthesis of biliverdin IXα (BV) and chlorophyll (Chl). Known sites of BV reduction by FDBRs are indicated. (B) Absorption spectra of cyanobacterial pigments in isolated protein complexes (Chl+carotenoids, green; PCB, blue; PEB, pink). (C) Reduction of BV by previously described FDBRs. Bilin binding to different proteins is indicated. Dashed lines, FDBRs not found in cyanobacterial genomes. (D) Known 4-electron reductions by FDBRs, including partial formation of PEB by pre-3 (this work). BV, biliverdin IXα; DHBV, dihydrobiliverdin; PΦB, phytochromobilin; PEB, phycoerythrobilin; PUB, phycourobilin; PCB, phycocyanobilin.

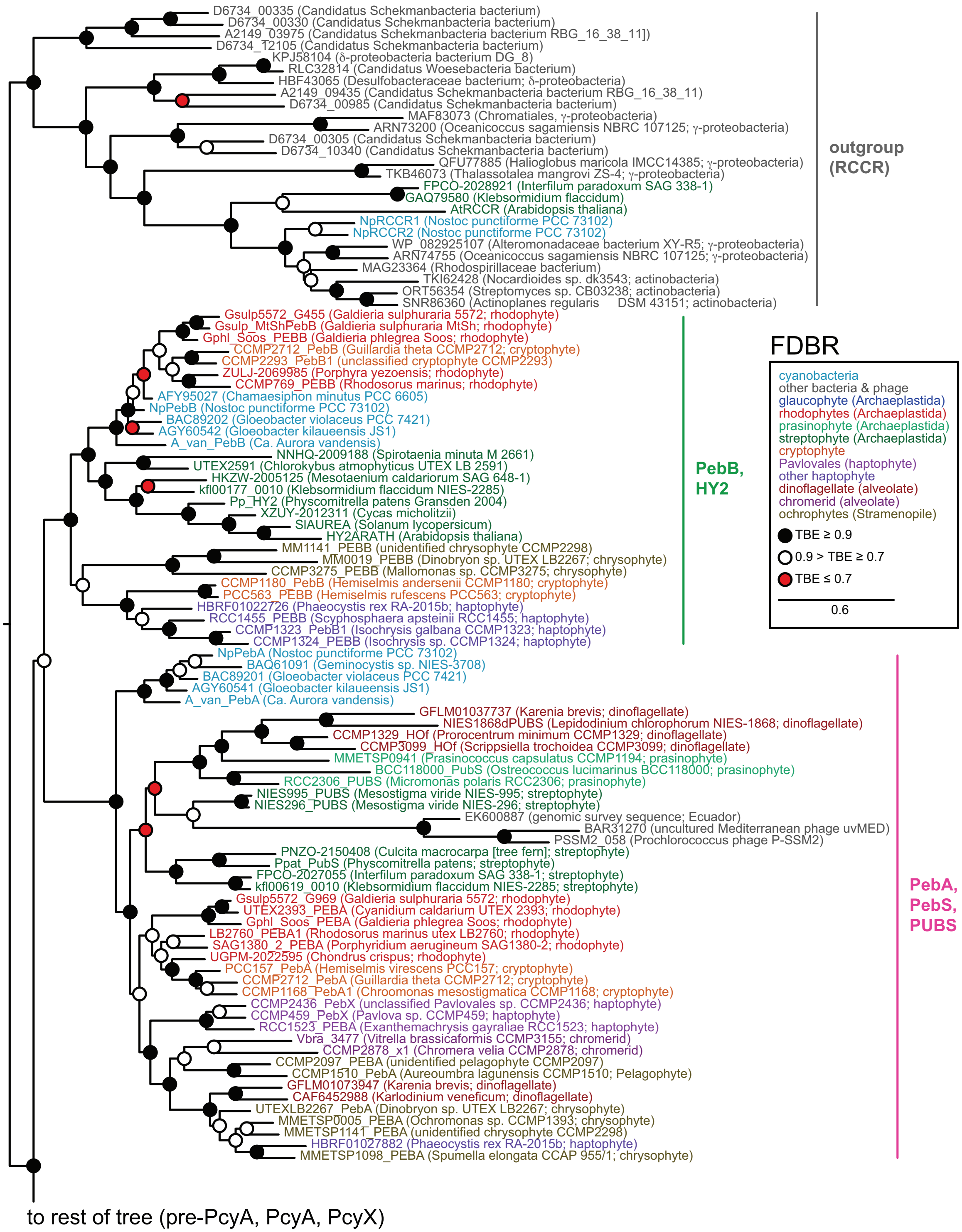

Fig. S2. Phylogenetic analysis of FDBRs. The outgroup, PebA/PebS/PUBS, and PebB/HY2 lineages of the full FDBR tree from Fig. 1 are shown in detail.

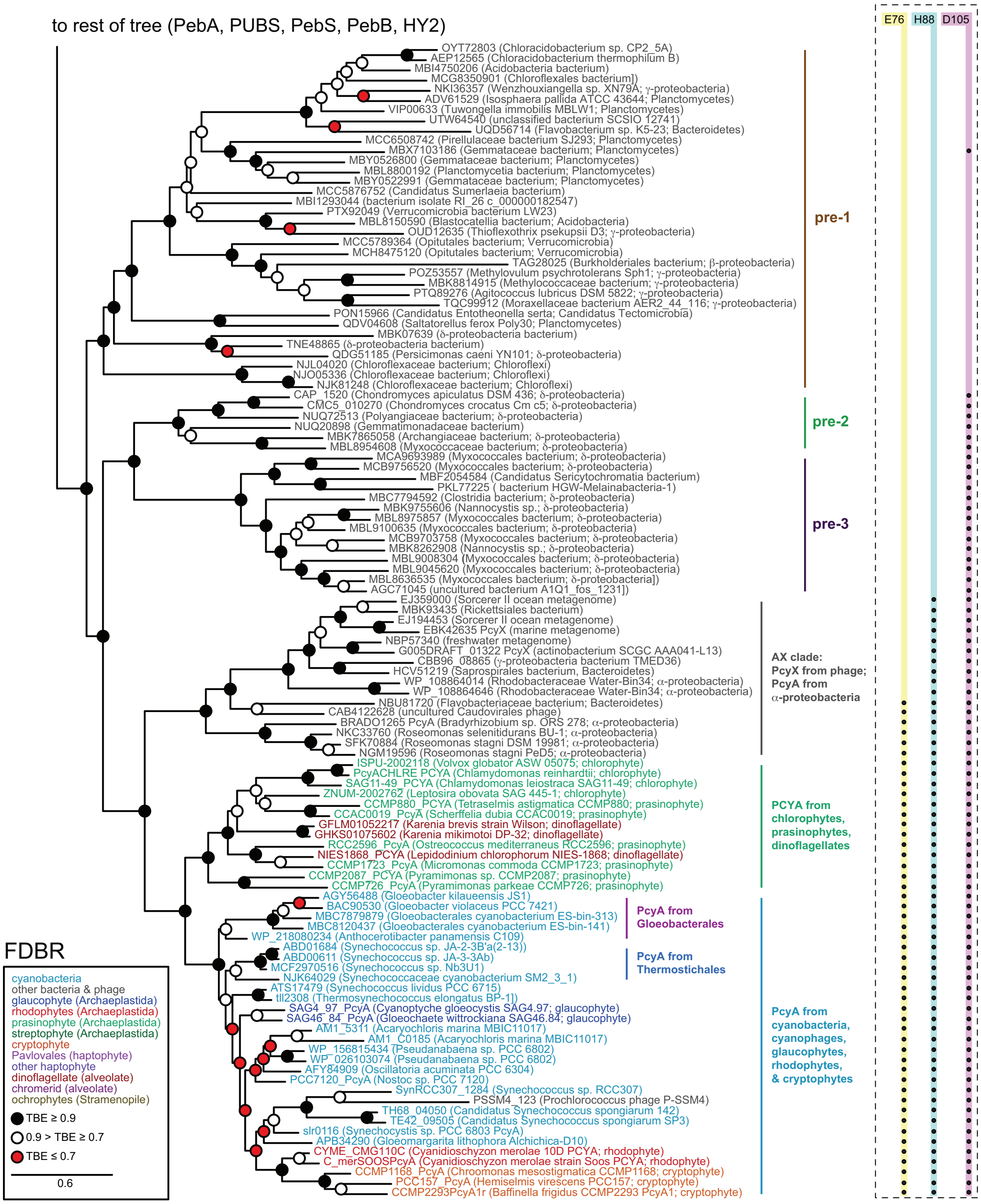

Fig. S3. Phylogenetic analysis of FDBRs. The pre-PcyA/PcyA lineage of the full FDBR tree from Fig. 1 is shown. The presence of Glu76, His88, and Asp105, three residues are implicated in conversion of BV to PCB by PcyA (7, 8), is indicated.

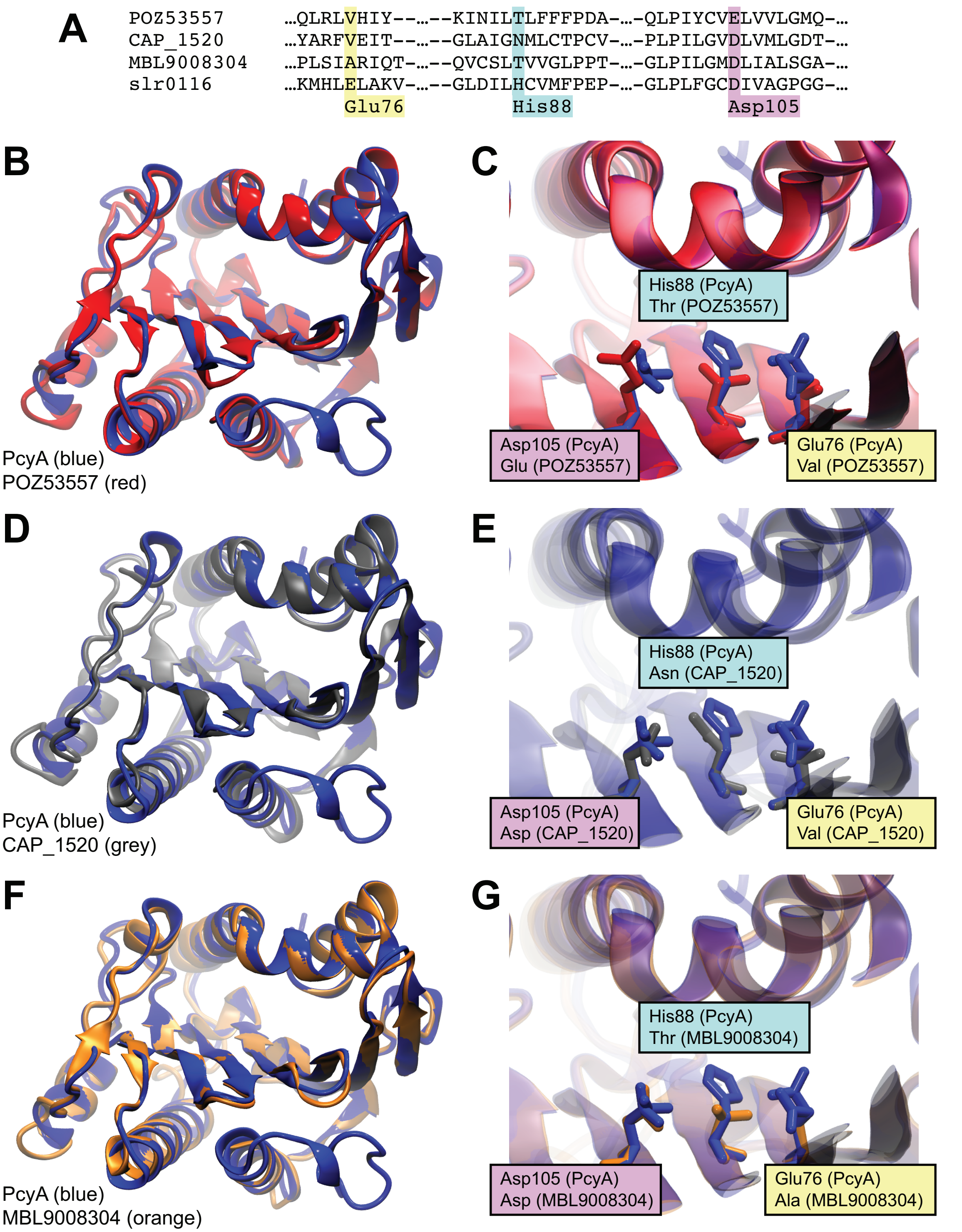

Fig. S4. Homology modeling of characterized pre-PcyA proteins. (A) Parts of the multiple sequence alignment of FDBRs are shown for *Synechocystis* PcyA (slr0116) and pre-PcyA proteins studied in this work. Catalytic residues of PcyA are highlighted using the color scheme of Fig. S3. (B) A homology model for POZ53557 (red) is superimposed on the experimental structure of PcyA with bound BV (blue; PDB accession 2D1E, (9)), showing conservation of the overall fold. (C) A detail view of potential catalytic residues of POZ53557. (D) A homology model of CAP_1520 (grey) is compared to PcyA. (E) A detail view is shown for CAP_1520. (F) A homology model of MBL9008304 (orange) is compared to PcyA. (G) A detail view is shown for MBL9008304.

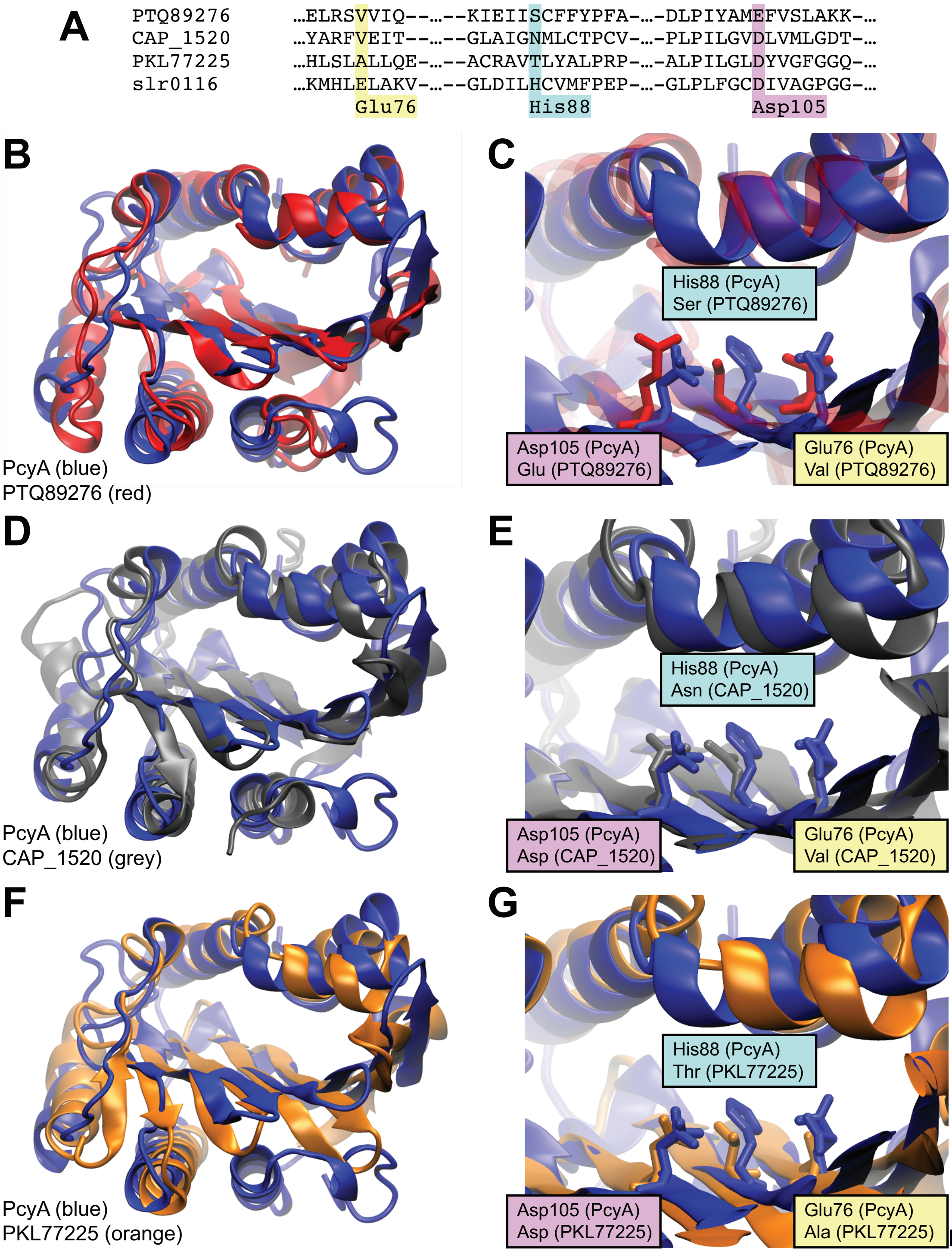

Fig. S5. Active-site residues in pre-PcyA predictions from the AlphaFold database. (A) Parts of the multiple sequence alignment of FDBRs are shown for *Synechocystis* PcyA (slr0116) and pre-PcyA proteins PTQ89276, CAP_1520, and PKL77225, for which predicted structures generated by AlphaFold (10) are available online (alphafold.ebi.ac.uk). Positions corresponding to catalytic residues of PcyA are highlighted using the color scheme of Fig. S3. (B) The predicted structure for PTQ89276 is superimposed on the experimental structure of PcyA. (C) A detail view is shown. (D) The overall fold of the predicted structure for CAP_1520 is shown. (E) A detail view is shown. (F) The overall fold of the predicted structure for PKL77225 is shown. (G) A detail view is shown.

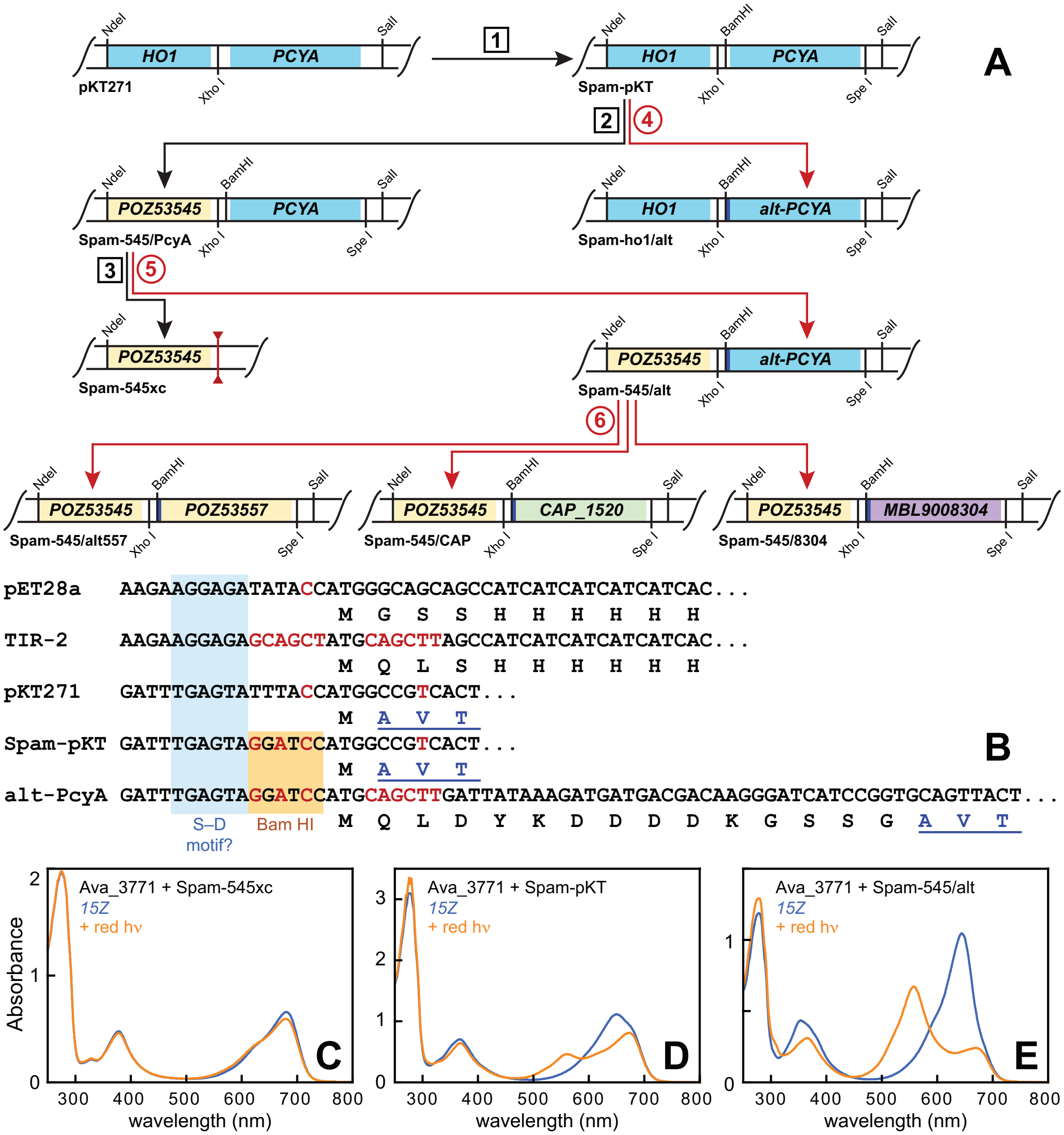

Fig. S6. Reconstitution of FDBR activity in *E. coli.* (A) Plasmids and relevant restriction sites are shown. Steps 1-3 (black squares) have been previously published (4) and are included for clarity; new steps are indicated with red circles and are discussed in the Methods. (B) The alt-PcyA 5' end is shown with pKT271, Spam-pKT, pET28a, and the recently published TIR-2 derivative of pET28a (5). Noncanonical Shine-Dalgarno sequences are indicated (blue box, S-D motif), as is the BamHI site in orange. Homology to the TIR-2 sequence is indicated with red bases. The alt-PcyA 5' end also incorporates a FLAG tag and a short linker. N-terminal sequence of PcyA (blue) is underlined. (C-E) Absorption spectra are shown for CBCR Ava_3771 (6) after co-expression with Spam-545xc (C, undiluted), Spam-pKT (D), and Spam-545/alt (E, at 1:1 dilution).

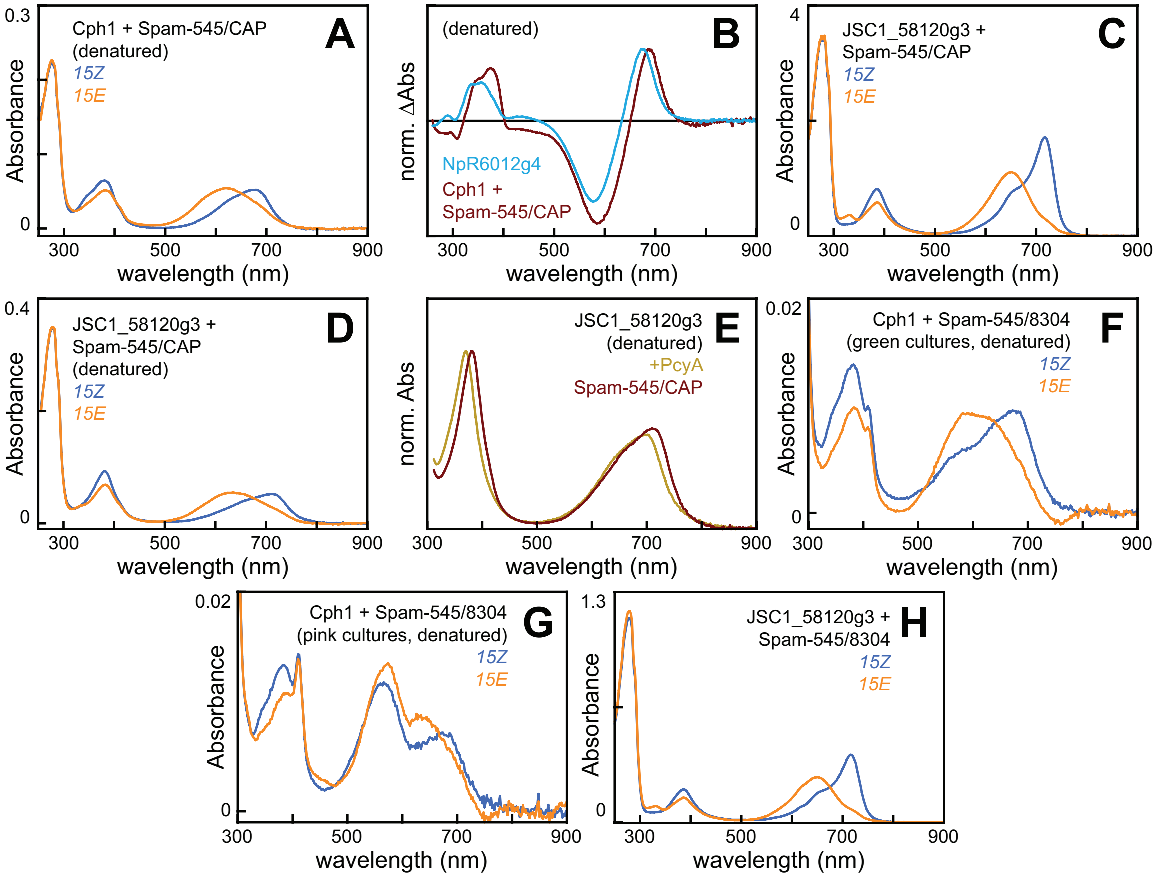

Fig. S7. Characterization of pre-PcyA enzymes using recombinant bilin-binding proteins. (A) Absorption spectra are shown for denatured Cph1 after co-expression with Spam-545/CAP (HO POZ53545 and FDBR CAP_1520). (B) Normalized photochemical difference spectra are shown for denatured Cph1 with Spam-545/CAP (brick red) and CBCR NpR6012g4 with PcyA (cyan). (C) Absorption spectra are shown for native CBCR JSC1_58120g3 after co-expression with Spam-545/CAP. (D) Absorption spectra are shown for the same material after denaturation. (E) Normalized absorption spectra are shown for denatured *15Z* JSC1_58120g3 with PcyA (bronze) or Spam-545/CAP (brick red). (F) Absorption spectra are shown for denatured Cph1 from green cultures after co-expression with Spam-545/8304 (HO POZ53545 and FDBR MBL9008304). (G) Absorption spectra are shown for denatured Cph1 from pink cultures after co-expression with Spam-545/8304. (H) Absorption spectra are shown for native JSC1_58120g3 after co-expression with Spam-545/8304.

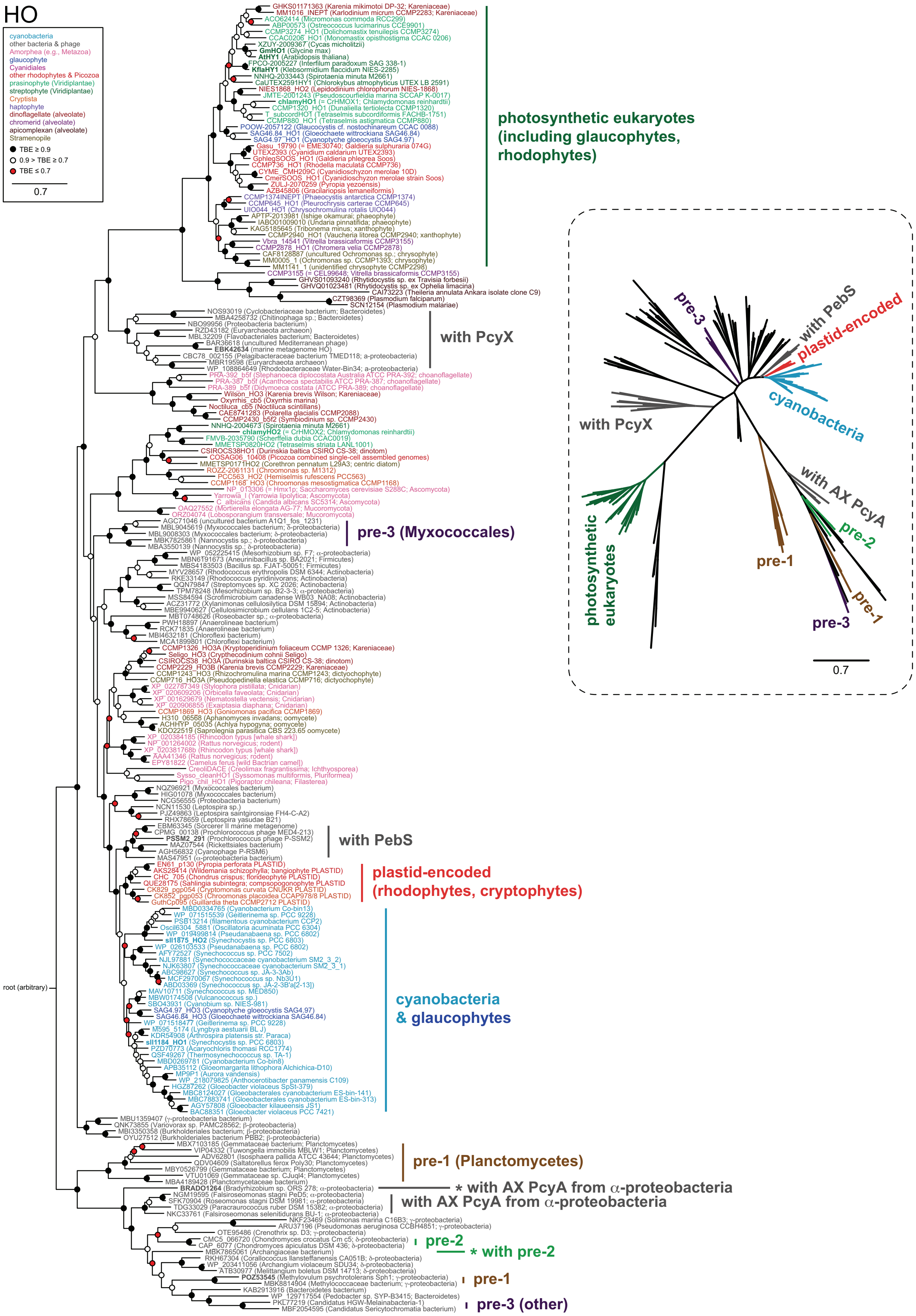

Fig. S8. Phylogenetic analysis of HO. The full tree is shown for heme oxygenase (HO). Root placement is arbitrary. *(inset)* The same tree is shown in unrooted form.

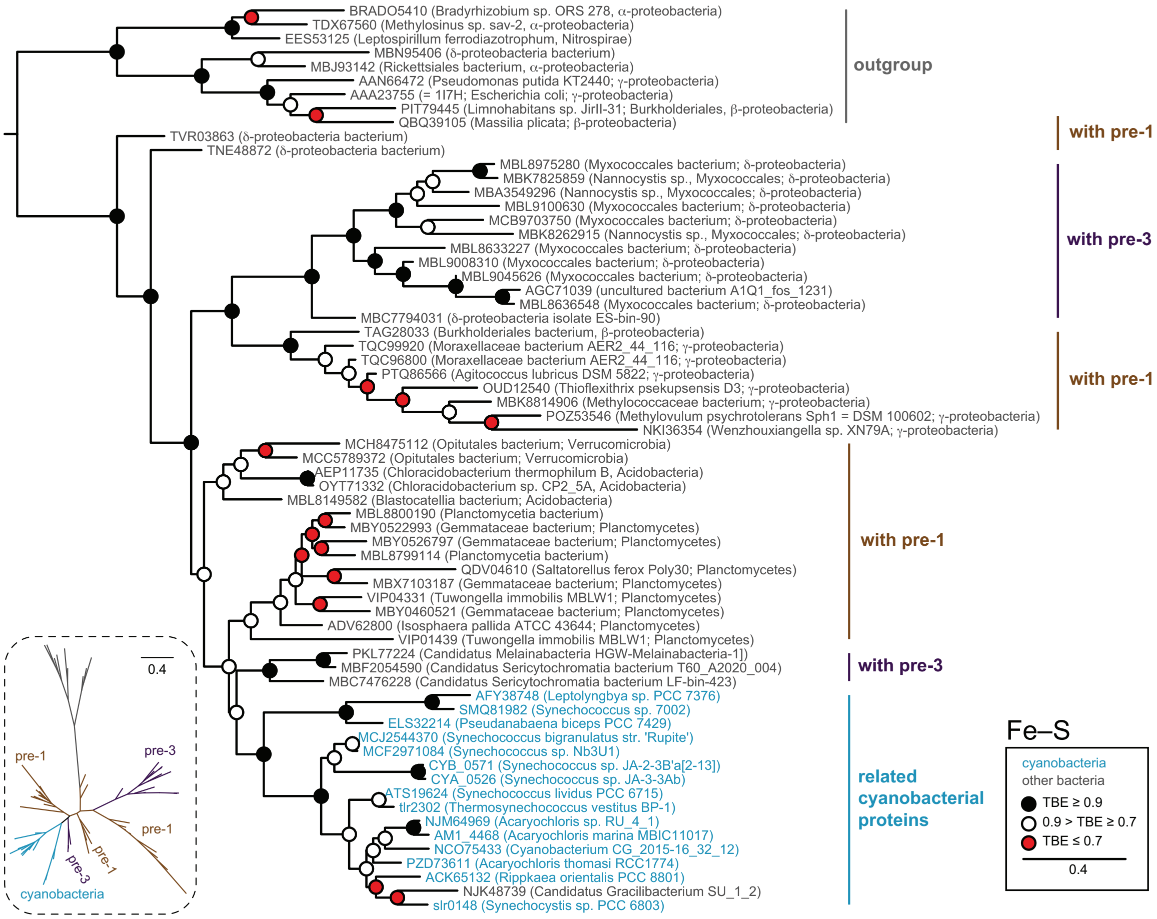

Fig. S9. Phylogenetic analyses of Fe–S proteins. The full maximum-likelihood tree is shown for Fe–S proteins associated with pre-PcyA sequences. *(inset)* The same tree is shown in unrooted form.

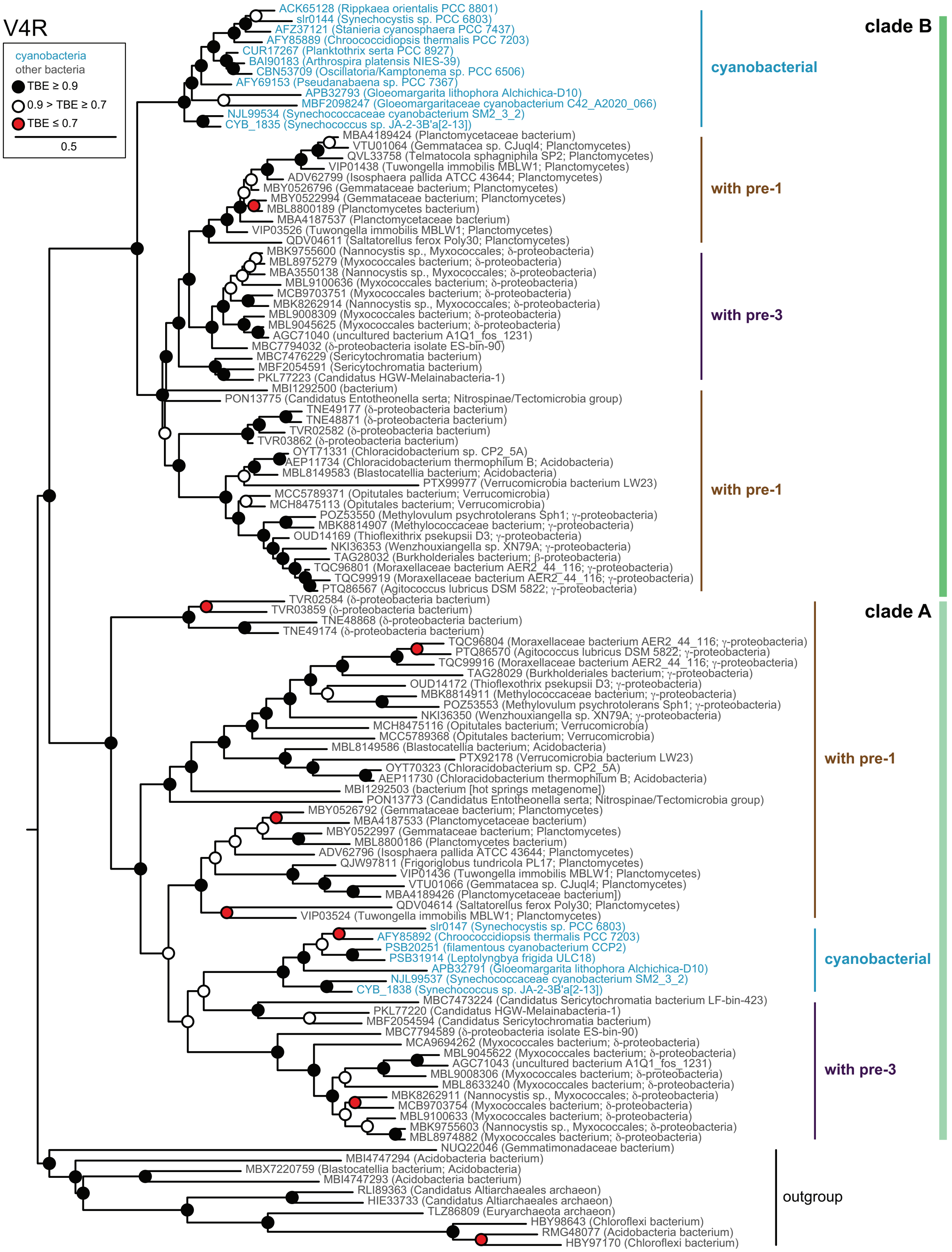

Fig. S10. Phylogenetic analysis of V4R proteins. The full maximum-likelihood tree is shown for V4R-domain-containing proteins. Two clades associated with pre-PcyA sequences are indicated in the color scheme of Fig. 3A. (inset) The same tree is shown in unrooted form.

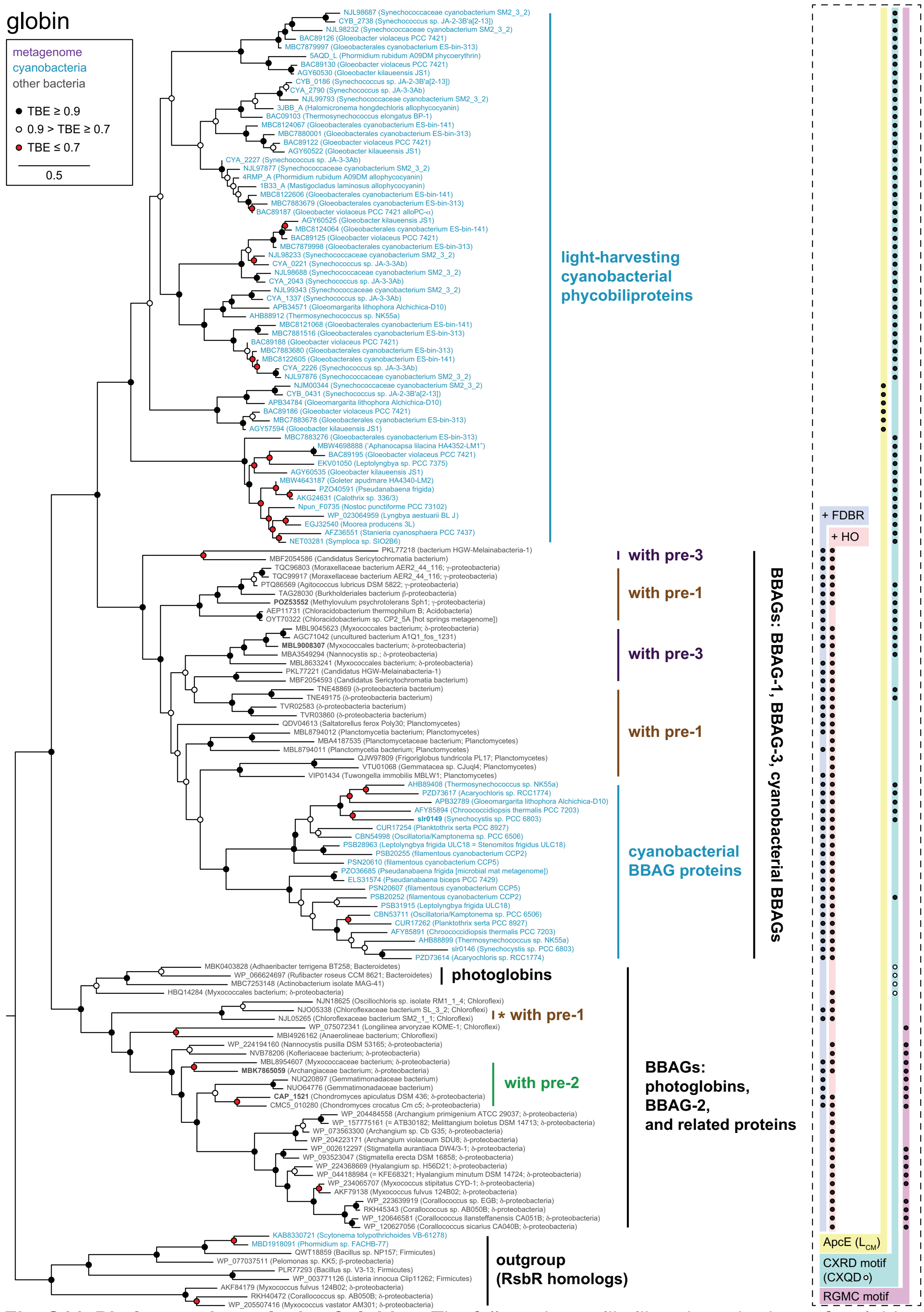

Fig. S11. Phylogenetic analysis of globins. The full maximum-likelihood tree is shown for globins

(Fig. 5). *(right inset)* The association of BBAGs with FDBRs and HO and the presence of various Cys-containing motifs in BBAGs and phycobiliproteins are indicated.

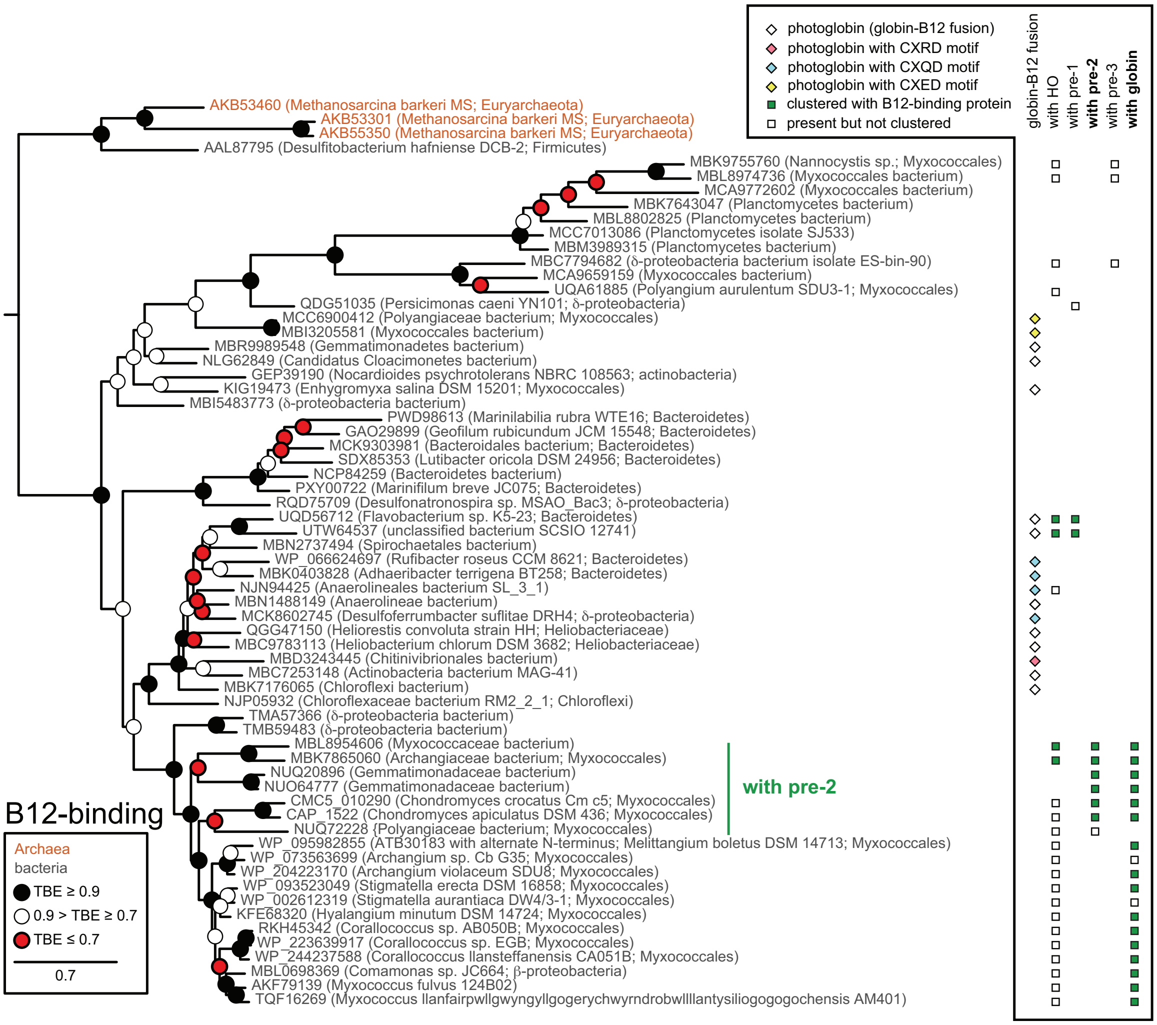

Fig. S12. Phylogenetic analysis of B12-binding proteins. The full maximum-likelihood tree is shown for B12-binding proteins associated with pre-2 and related proteins. (*right-hand inset*) The co-occurrence of B12-proteins with HO, pre-PcyA lineages, and BBAGs is shown, as is the presence of Cys-containing motifs (potential chromophorylation sites) in globin-B12 fusions (photoglobins (11)).

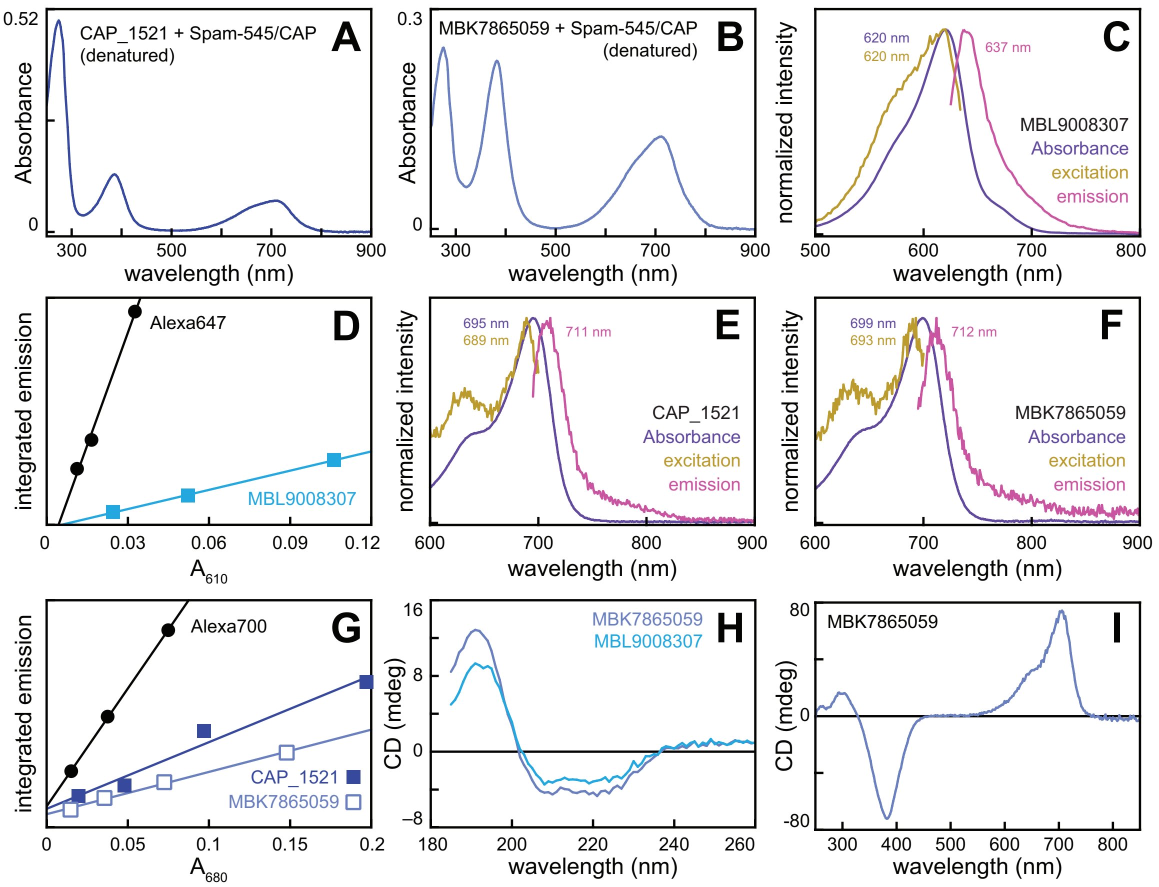

Fig. S13. Characterization of recombinant BBAGs. (A) The absorption spectrum is shown for denatured BBAG-2 CAP_1521 after co-expression with Spam-545/CAP (HO POZ53545 and FDBR CAP_1520). (B) The absorption spectrum is shown for denatured BBAG-2 MBK7865059 after co-expression with Spam-545/CAP. (C) Normalized absorption (purple), excitation (bronze; λ_{em} 665 nm), and emission (dusty rose; λ_{ex} 610 nm) spectra are shown for BBAG-3 MBL9008307 with PCB. Peak wavelengths are indicated. (D) Integrated fluorescence emission is plotted against absorbance at 610 nm for Alexa647 (black filled circles; r^2 = 0.996) and MBL9008307 (cyan filled squares; r^2 = 0.999). The slope of the linear fit is proportional to the fluorescence quantum yield. (E) Normalized absorption (purple), excitation (bronze; λ_{em} 715 nm), and emission (dusty rose; λ_{ex} 680 nm) spectra are shown for CAP_1521. Peak wavelengths are indicated. (F) Normalized absorption (purple), excitation (bronze; λ_{em} 715 nm), and emission (dusty rose; λ_{ex} 680 nm) spectra are shown for MBK7865059. Peak wavelengths are indicated. (G) Integrated fluorescence emission is plotted against absorbance at 680 nm as in panel D for Alexa700 (black filled circles; r^2 > 0.999), CAP_1521 (dark blue filled squares, r^2 = 0.968), and MBK7865059 (periwinkle open squares; r^2 = 0.996). (H) CD spectra are shown for CAP_1521 (cyan) and MBK7865059 (periwinkle) in the UV region. (I) The CD spectrum is shown for MBK7865059 in the bilin region.

Table S1. Spectroscopic parameters for recombinant bilin-binding proteins[a]

Protein	Bilin synthesis plasmid	SAR	denatured *15Z* λ_{max}	*15Z* λ_{max}	*15E* λ_{min}
Ava_3771	Spam-545xc	0.3	708; 380	684	598
Ava_3771	pKT271[b]	0.4	670; 370	646	554
Ava_3771	Spam-pKT	0.4	678; 370	644	554
Ava_3771	Spam-545/PcyA	0.7	674; 370	644	554
Ava_3771	Spam-ho1/alt	0.7	672; 370	644	556
Ava_3771	Spam-545/alt	0.9	670; 364	644	554
NpR6012g4	pPL-PCB	1.6	666; 356	652	542
Cph1-N514	pKT271[c]	0.2	664; 360	660	704
Cph1-N514	Spam-545/alt557	0.03	not done	672	724
Cph1-N514	Spam-545/CAP	0.6	674; 378	672	722
Cph1-N514	Spam-545/8304 (green)	0.2	672; 382	670	720
Cph1-N514	Spam-545/8304 (pink)	0.06	566; 382	670	720
JSC1_58120g3	Spam-545/CAP	0.5	710; 382	718	628
JSC1_58120g3	Spam-545/8304	0.3	712; 382	716	630
JSC1_58120g3	pPL-PΦB[d]	0.9	704; 380	724	628
POZ53552	Spam-545xc	0.1	694; 380	680	None
POZ53552	Spam-545/alt557	0.1	706; 380	680	None
POZ53552	Spam-545/CAP	0.1	676; 380	684	None
POZ53552	Spam-545/8304	0.04, 0.3	678, 564; 378	684, 568	None
MBL9008307	Spam-545/alt	1.2	664; 358	620	None
MBL9008307	Spam-545/8304	0.4, 0.7	670, 564; 378	644, 560	None
CAP_1521	Spam-545/CAP	0.6	708; 386	696	None
MBK7865059	Spam-545/CAP	1.6	712; 382	700	None

[a]All wavelengths are in nm. Specific absorbance ratio (SAR) was calculated as peak absorbance for the 15Z red band divided by that for the 280 nm band. Peak wavelengths for denatured samples and BBAGs are from static spectra. Those for phytochromes and CBCRs are for difference spectra.

[b]Data from (6).

[c]Data from (12).

[d]Data from (13).

Table S2: Statistics for phylogenetic analyses

Protein family	Presentation	Sequences	Characters	*n* < 90% complete
FDBR	Figs. 1, S2-S3	212	177	0
HO	Fig. S8	206	170	4
Fe–S	Fig. S9	65	101	0
V4R	Fig. S10	118	183	0
globin	Figs. 5, S11	150	112	3
catenated	Fig. 4	38	890	0
B12-binding	Fig. S12	64	193	0

SI References

1. F. Lemoine *et al.*, Renewing Felsenstein's phylogenetic bootstrap in the era of big data. *Nature* **556**, 452-456 (2018).
2. N. C. Rockwell, J. C. Lagarias, Flexible mapping of homology onto structure with homolmapper. *BMC Bioinformatics* **8**, 123 (2007).
3. K. Mukougawa, H. Kanamoto, T. Kobayashi, A. Yokota, T. Kohchi, Metabolic engineering to produce phytochromes with phytochromobilin, phycocyanobilin, or phycoerythrobilin chromophore in *Escherichia coli*. *FEBS Lett.* **580**, 1333-1338 (2006).
4. N. C. Rockwell, M. V. Moreno, S. S. Martin, J. C. Lagarias, Protein-chromophore interactions controlling photoisomerization in red/green cyanobacteriochromes. *Photochem. Photobiol. Sci.* **21**, 471-491 (2022).
5. P. J. Shilling *et al.*, Improved designs for pET expression plasmids increase protein production yield in Escherichia coli. *Commun. Biol.* **3**, 214 (2020).
6. N. C. Rockwell, S. S. Martin, F. Gan, D. A. Bryant, J. C. Lagarias, NpR3784 is the prototype for a distinctive group of red/green cyanobacteriochromes using alternative Phe residues for photoproduct tuning. *Photochem. Photobiol. Sci.* **14**, 258-269 (2015).
7. Y. Hagiwara *et al.*, Structural insights into vinyl reduction regiospecificity of phycocyanobilin:ferredoxin oxidoreductase (PcyA). *J. Biol. Chem.* **285**, 1000-1007 (2010).
8. M. Unno *et al.*, Insights into the Proton Transfer Mechanism of a Bilin Reductase PcyA Following Neutron Crystallography. *J. Am. Chem. Soc.* **137**, 5452-5460 (2015).
9. Y. Hagiwara, M. Sugishima, Y. Takahashi, K. Fukuyama, Crystal structure of phycocyanobilin:ferredoxin oxidoreductase in complex with biliverdin IXalpha, a key enzyme in the biosynthesis of phycocyanobilin. *Proc. Natl. Acad. Sci. U. S. A.* **103**, 27-32 (2006).
10. J. Jumper *et al.*, Highly accurate protein structure prediction with AlphaFold. *Nature* **596**, 583-589 (2021).
11. T. Schneider, Y. Tan, H. Li, J. S. Fisher, D. Zhang, Photoglobin, a distinct family of non-heme binding globins, defines a potential photosensor in prokaryotic signal transduction systems. *Comput. Struct. Biotechnol. J.* **20**, 261-273 (2022).
12. N. C. Rockwell, S. S. Martin, F. W. Li, S. Mathews, J. C. Lagarias, The phycocyanobilin chromophore of streptophyte algal phytochromes is synthesized by HY2. *New Phytol.* **214**, 1145-1157 (2017).
13. M. V. Moreno, N. C. Rockwell, M. Mora, A. J. Fisher, J. C. Lagarias, A far-red cyanobacteriochrome lineage specific for verdins. *Proc. Natl. Acad. Sci. U. S. A.* **117**, 27962-27970 (2020).
